# Competitive fitness of *Staphylococcus aureus* against nasal commensals depends on biotin biosynthesis and acquisition

**DOI:** 10.1093/ismejo/wraf248

**Published:** 2025-11-04

**Authors:** Kevser Bilici, David Gerlach, Laura Camus, Simon Heilbronner

**Affiliations:** Interfaculty Institute of Microbiology and Infection Medicine Institute for Medical Microbiology and Hygiene, UKT, 72076 Tübingen, Germany; Cluster of Excellence EXC 2124 Controlling Microbes to Fight Infections, 72076 Tübingen, Germany; Faculty of Biology, Microbiology, Ludwig-Maximilians-Universität München, 82152 Martinsried, Germany; Faculty of Biology, Microbiology, Ludwig-Maximilians-Universität München, 82152 Martinsried, Germany; Interfaculty Institute of Microbiology and Infection Medicine Institute for Medical Microbiology and Hygiene, UKT, 72076 Tübingen, Germany; Cluster of Excellence EXC 2124 Controlling Microbes to Fight Infections, 72076 Tübingen, Germany; Cluster of Excellence EXC 2124 Controlling Microbes to Fight Infections, 72076 Tübingen, Germany; Faculty of Biology, Microbiology, Ludwig-Maximilians-Universität München, 82152 Martinsried, Germany; German Center for Infection Research “DZIF” Partnersite Tübingen, 72076 Tübingen, Germany

**Keywords:** *S. aureus*, biotin, nasal colonisation, nasal microbiome, competitive fitness

## Abstract

The human nasal microbiome can serve as a reservoir for pathogens. In particular, the opportunistic pathogen *Staphylococcus aureus* can be a member of the nasal microbiome increasing the risk of subsequent infections. The nasal carriage of *S. aureus* is known to be positively and negatively impacted by nonpathogenic species, suggesting interactions between the pathogen and commensals, but the underlying molecular mechanism remains largely unclear. Herein we demonstrate that *S. aureus* competes with nasal commensals for the coenzyme biotin. Biotin is crucial for all living organisms and we show that depletion of biotin impairs *S. aureus* growth and membrane integrity. We found the nasal cavity to be a biotin-limited environment, suggesting competition for the coenzyme within the microbiome. For some nasal commensals and *S. aureus,* we observed biotin prototrophy and all strains released biotin into the environment. In contrast, other commensals and especially coagulase-negative staphylococci (CoNS) were found to be biotin auxotrophs and strongly reliant on prototrophic strains under biotin-limited conditions. We show that high-affinity biotin uptake systems are used by prototrophic and auxotrophic strains alike and represent crucial factors to optimize competitive fitness of species in co-culture. Together, our data show that biotin-mediated interactions occur between the species of the human nasal microbiome and provide evidence for interspecies competition and co-dependency.

## Graphical Abstract

**Graphical Abstract:**
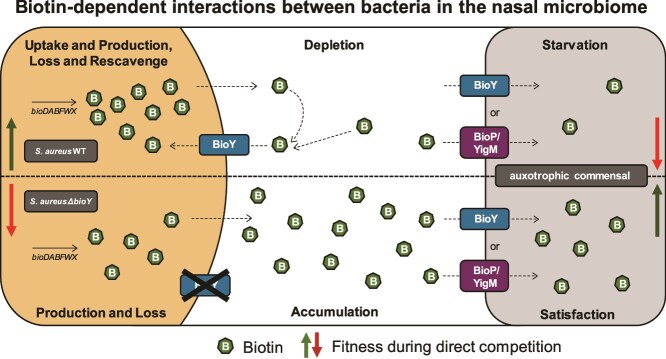

## Introduction

*Staphylococcus aureus* is an important human pathogen and causes superficial skin and soft tissue infections as well as life threatening invasive diseases such as sepsis, meningitis, pneumonia, and endocarditis [1]. As an opportunistic pathogen, *S. aureus* colonizes the anterior nares of ~30% of the human population [1–3]. A link between *S. aureus* nasal carriage and development of subsequent infection by the same strain is well known [4], but molecular reasons impacting carriage status remain largely unknown. However evidence is gathering that interactions with the nasal microbiome can be of critical importance [5–13]. For example, *Corynebacterium* spp, *S. epidermidis,* and *S. lugdunensis* can antagonize *S. aureus* colonization [6, 9, 13, 14]. Some antagonizing interactions are caused by direct or indirect inhibition by antimicrobial compounds [14–17] while others remain poorly understood and seem more complex.

The human nasal cavity is a low-nutrient environment. Metabolomic analysis has shown, that nasal secretions contain low concentrations of mono- and disaccharides as well as of amino acids [18]. Trace metals are limited due to the secretion of chelating molecules [19, 20] while other trace nutrients such as vitamins were not detectable [18]. This suggests two possible interactions at the interface of the nasal microbiome and the nasal environment. Firstly, nutritional limitation might trigger strict competition for limited, essential nutrients or, secondly, bacteria might cooperate and engage in cross-feeding interactions by sharing public goods [21]. It is not known if such interactions occur between *S. aureus* and nasal commensals and involved nutrients as well as the effects of the interactions on *S. aureus* are unclear.

**Figure 1:**
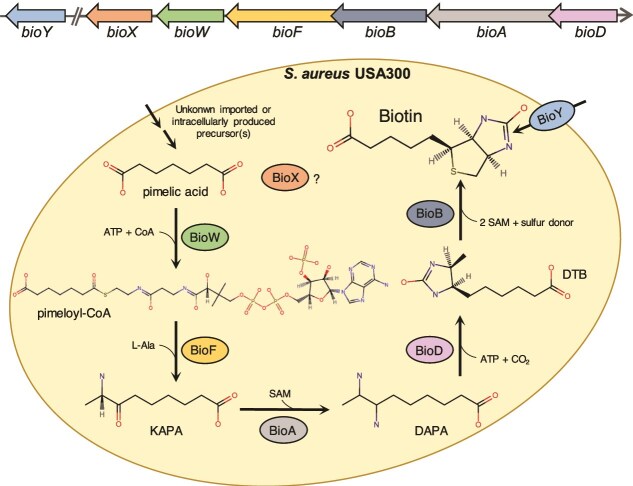
Schematic diagram of biotin biosynthesis and transport in *S. aureus* USA300. Genes and corresponding enzymatic functions are indicated. Briefly, pimelic acid is converted to pimeloyl-CoA by BioW (pimeloyl-CoA synthase, SAUSA300_2369). BioX (SAUSA_2368) may act as an acyl carrier protein involved in the synthesis of pimeloyl-CoA. BioF (KAPA synthase, SAUSA300_2370) mediates the reaction to 7-keto-8-aminopelarogonic acid (KAPA) which subsequently undergoes transamination reaction by BioA (DAPA aminotransferase, SAUSA300_2372) to form 7,8-diaminopelargonic acid (DAPA). Following the synthesis of dethibiotin (DTB) by BioD (dethibiotin synthase, SAUSA300_2373), biotin is formed using the biotin synthase BioB (SAUSA300_2371). In addition, biotin can be transported by the ECF-type transporter BioY (SAUSA300_2233). Chemical structures were created by using the InChl from PubChem and the chemical sketch tool of PDB.

Vitamins are micronutrients that are crucial for the metabolism of all living organisms. In particular, vitamins of the water soluble B-group serve as cofactors in different enzymatic pathways needed for proliferation of microorganisms [22, 23]. Biotin (vitamin B7) is an essential cofactor for enzymes catalyzing carboxylation, decarboxylation, and transcarboxylation reactions needed in different metabolic pathways, including gluconeogenesis, fatty acid synthesis, and amino acid catabolism [24]. Plants, some prokaryotes and fungi are able to synthesize biotin *de novo* while humans are auxotrophic. In bacteria four conserved enzymes BioF, BioA, BioD, and BioB are needed to produce biotin from pimeloyl-CoA. Depending on the organism, the pimeloyl moiety is provided by different pathways, needing different additional proteins BioW and BioI [25–27] or BioC and BioH [28–30] or BioZ [31]. *S. aureus* is biotin prototrophic and possesses the biotin operon *bioDABFWX* (Fig. 1). In brief, pimelic acid is converted to pimeloyl-CoA by BioW (pimeloyl-CoA synthase). At this point, BioX may also act as an acyl carrier protein involved in the synthesis of pimeloyl-CoA [25]. BioF (KAPA synthase) then mediates the reaction to 7-keto-8-aminopelarogonic acid (KAPA) with a subsequent reaction to 7,8-diaminopelargonic acid (DAPA) directed by BioA (DAPA aminotransferase). Following the synthesis of dethibiotin (DTB) by BioD (dethibiotin synthase) biotin is formed using the biotin synthase BioB. Biotin biosynthesis is a metabolically costly pathway because at least six enzymes and 19–20 molecules of ATP equivalents are needed to produce one molecule of biotin [32, 33]. The last reaction of biotin biosynthesis namely, the cleavage of two S-adenosyl-methionine (SAM) by BioB to form the tetrahydrothiophene ring is the main energy consuming reaction, needing six ATP equivalents [33].

In addition to *de novo* synthesis, bacteria are able to acquire biotin from the environment using the biotin transporters BioY or YigM/BioP. BioY is the substrate-binding component (S component) of an energy-coupling factor (ECF)-type ABC transporter. The *bioY* gene, usually occurs in an operon with the genes encoding the transmembrane protein (T component), and the homo- or hetero-oligomeric ATPases (A component) [34, 35]. ECF-type transporter are known to be high-affinity micronutrient uptake systems in prokaryotes with affinities in the low nanomolar to picomolar range [35]. Thereby ECF-type transporters allow the smallest traces of costly metabolites to be scavenged [35]. YigM/BioP belongs to the carboxylate/amino acid/amine family of secondary active transporters, which are mainly found in *Gammaproteobacteria* and *Epsilonproteobacteria* [36–39]. *Escherichia coli* and *S. aureus* are known to be prototrophic but also to import biotin from the environment via YigM/BioP and BioY, respectively [40].

The importance of biotin biosynthesis and uptake for *S. aureus* in the context of nutritional limitation and microbial competition within the nasal cavity remains unclear. Herein we show biotin availability to be crucial for *S. aureus* growth and membrane integrity. *S. aureus* as well as several nasal commensals produce and release biotin to the environment. In contrast, other commensals and especially coagulase-negative staphylococci (CoNS) were found to be biotin auxotrophic and profited strongly from the presence of biotin prototrophic strains in a biotin-limited environment. High-affinity biotin uptake systems were used by prototrophic and auxotrophic strains alike and mediated direct competition for biotin between the members of the nasal microbiome.

## Material and methods

### Chemicals

If not stated otherwise, reagents were purchased from Sigma.

### Bacterial strains and growth conditions

Bacterial strains are listed in Suppl. Tab. S1. Depending on the experiment, overnight cultures were performed in either TSB (Oxoid, CM0129T) or BHI containing 0.2% Tween80 (BHI-T) at 37°C with agitation. Tetracycline (12.5 μg/ml) or chloramphenicol (10 μg/ml) was added where needed to maintain plasmids pC183-S3 and pIMAY, respectively (Suppl. Tab. S1/S2).

### Creation of marker less deletion mutants and chromosomal complementation

Deletion mutants in *S. aureus* USA300 JE2 were created as previously described [41]. In brief: For *bioA* and *bioY*, 500 bp flanking regions upstream and downstream of the genes were amplified from chromosomal DNA by using A/B and C/D primers (Suppl. Tab. S2) with overlapping sequences. A 1-kb deletion fragment with an ATG-TAA scar was created using spliced overlap extension PCR (SOE-PCR), cloned into pIMAY by restriction digestion and used to transform *S. aureus* USA300 JE2. Mutants were created using allelic exchange as described [41].

For genomic complementation, a silent mutation creating a restriction site (*BamHI* for *bioA* and *SmaI* for *bioY*) was introduced into the wildtype allele by SOE-PCR. The constructs were cloned into pIMAY and used for reinsertion of the allele into the mutants by allelic replacement.

For creating fluorescent *S. aureus* strains, the GFP-expressing plasmid pC183-S3 was used to transform *S. aureus* using standard procedure.

All used oligonucleotides and plasmids are summarized in Suppl. Tab. S2.

### Streptavidin-treated tris minimal succinate medium and agar

Tris minimal succinate medium (TMS) was prepared as previously described [42] with the exception of adding 10 g CAS amino acids (Bacto, Gibco Thermo Fisher, 223050) and 27,67 g Di-sodium succinate hexahydrate (Merck, 8.20151.0500). After autoclaving the TMS medium, 0.01% (v/v) Streptavidin agarose (Merck, 69203) was added to the medium and stirred overnight at 4°C for at least 18 h. The medium was sterile filtrated on the next day by using a Nalgene Rapid-Flow filter from Thermo Scientific (0.2 μm). After sterile filtration following chemicals were added to 1 l Streptavidin-treated TMS medium (sTMS): 1 ml of 1 M MgCl_2_ (Sigma Aldrich, M8266-100G), 1 ml of 0.1 M CaCl_2_ (Sigma Aldrich, C5670-100G), 1 ml of 22 mg/ml L-cysteine (Sigma-Aldrich, 1.02838.0100), and 2 ml of 10 mg/ml L-tryptophan (Sigma-Aldrich, 93 659-50G). After adding the supplements, the medium was stored at 4°C. A vitamin stock was prepared by adding 25 μl of 5 mg/ml cyanocobalamin (Merck, 5.24950.0010), 5 μl of 20 mg/ml 4-aminobenzic acid (Sigma Aldrich, A9878-25G), 6.25 μl of 40 mg/ml nicotinic acid (Merck, 72 309-100G), 6.25 μl of 20 mg/ml D-pantothenic acid hemicalcium salt (Merck, 21 210-5G-F), 7.5 μl of 50 mg/ml pyridoxamine dihydrochloride (Merck, 545 068-5GM), 5 μl of 50 mg/ml thiamine hydrochloride (Roth, T911.2), 5 μl of 50 mg/ml riboflavin (Sigma, R4500-5G) to 1187.5 μl ddH_2_O. The vitamin stock was stored at −20°C. On the day of experiment vitamins from vitamin stock were added freshly in a 1:1000 dilution to sTMS.

For preparing sTMS agar, TMS medium was prepared as described above with 1.5% European Agar (BD Diagnostic, 257 353). After autoclaving the TMS medium, 0.05 mg/ml Streptavidin (Roth, 6073.2), and all supplements (MgCl_2_, CaCl_2_, L-cysteine, L-tryptophane and vitamins, as mentioned above) were added and plates were poured. So freshly prepared agar was used for all experiments.

### Growth analysis in sTMS

Generation of the inoculum: Bacteria were grown to stationary phases overnight in either TSB (*S. aureus* USA300 JE2 and mutants) or BHI-T (all nasal isolates, *E. coli* BW25113 and *E. coli* BW25113 *yigM*::Kan) at 37°C with agitation. Cultures were centrifuged for 1 min at 15871 rcf and washed once with PBS. Cells were adjusted to OD_600_ = 0.1 and 1 μl was used to inoculate 1 ml of sTMS (final OD_600_ = 0.0001). Supplementation with biotin: When appropriate, final biotin concentrations (0015625 nM to 4 nM) were added using 1 μl or 1000-fold concentrated stock solutions to 1 ml of sTMS. For generation of spent medium, nasal bacteria were grown to stationary phase overnight in BHI-T at 37°C with agitation, harvested by centrifugation (15 871 rcf) and washed once with PBS. 10 ml sTMS medium were inoculated to an OD_600_ of 0.05 and grown for 24 h (stationary phase) at 37°C with agitation. Cells were removed by centrifugation (10 min at 3197 rcf), the supernatants were collected and sterile filtered using a 0.45 μM Millex-HA filter (SLHA033SB). Sterility was confirmed by plating of 200 μl on TSA plates and supernatants were stored until use at 4°C. Spent medium was diluted to 25% by mixing 250 μl with 750 μl fresh sTMS. Growth curve generation: After inoculation 500 μl of the sTMS was transferred to a 48-well microplate (Nunc, Thermo Scientific) and growth was monitored automatically by measuring the OD_600_ every 15 min for 25 h to 40 h in an Epoch2 reader (BioTek, Agilent) at appropriate temperatures (30°C, 37°C or 45°C) with double orbital shaking.

### Quantification of biotin levels

For biotin level determination the IDK Biotin ELISA-Kit from Immundiagnostik (K8141) was used according to the manufacturer instructions.

### Human and animal body fluids

Blood was drawn from healthy human volunteers as well as from cotton rats in accordance with ethical allowance from the legal authority in Tübingen (Project numbers 369/2018BO2 and IMIT 0/21 G). Human serum was gained by centrifugation (8000 rcf for 10 min) of blood drawn using heparinized tubes from three female and one male volunteer (23–45 years). Cotton rat plasma was obtained from fresh blood samples that were centrifuged (8000 rcf for 10 min).

Nasal secretions were collected from healthy human volunteers (25–45 years; two females and three males) in accordance with ethical allowance by the legal authority in Munich (Project Nr.: 24-0654). Flow of nasal secretions was induced by mild physical activity for 5 min in a −20°C room and collected by aspiration. The secretions were stored at −80°C and centrifuged (8000 rcf for 10 min) prior to use.

All human samples were taken with informed written consent.

### Crude cell extracts and streptavidin blot

For standard experiments under biotin-saturation, cultures were grown in either TSB (*S. aureus* USA300 JE2 and mutants) or BHI-T (all nasal isolates, *E. coli* BW25113 and *E. coli* BW25113 *yigM:*:Kan) and used directly. For experiments reflecting biotin-limited conditions, prototrophic strains were cultivated overnight in TSB/BHI-T, harvested by centrifugation (1 min at 15871 rcf) and washed once with PBS. Bacteria were adjusted to OD_600_ = 0.05 in 10 ml sTMS (in 100 ml flasks) and the cultures were grown for 24 h at 37°C with agitation. Overnight cultures (1 ml of OD_600_ = 1) from different media (TSB/BHI-T/sTMS) was harvested by centrifugation (1 min at 15871 rcf) and washed three times in PBS. The bacterial pellets were resuspended in 100 μl PBS and transferred to tubes containing glass beads (MN Bead Tubes Typ B, 740812.50). Cells were disrupted using FastPrep-24 (6.5 m/s for 60 sec) and centrifuged (1 min at 15871 rcf). Supernatants were transferred into fresh Eppendorf tubes and centrifuged again (1 min at 15871 rcf) to remove all remaining glass beads. 50 μl of the supernatant was mixed with 12.5 μl Lämmli buffer (BioRad, 1610747) and boiled for 10 min at 100°C in a heating block. 15 μl were loaded on two 13% SDS-PAGE (ROTIPHORESE Gel 30 (37,5:1)) gels. One gel was stained with BlueSafe (NZYtech, MB15201) as loading control and the other one was used for the streptavidin blot.

For the streptavidin blot, the proteins were blotted on a nitrocellulose membrane after separation using the semi-dry blotter Trans-Blot (BioRad). The membrane was incubated at room temperature in the dark in blocking buffer (Thermo Scientific, 37542) for 30 min on a shaking platform. The blocking buffer was discarded and the membrane was incubated with the streptavidin-AP conjugate (Merck/Roche, 11089161001) diluted 1:1000 in 10 ml blocking buffer and incubated as before. The membrane was washed twice in TBS-T (Tris-buffered saline with Tween20, Roth, 1061.1) for 5 min and once with TBS (Tris-buffered saline, Roth, 1060.1) at RT and in the dark. To detect alkaline phosphatase activity a staining solution with 1 ml 0.1% NBT (Biomol, 06428.1) and 9 ml Na_2_CO_3_ buffer (9.2 mM Na_2_CO_3_, 40 mM Na_2_CO_3_, pH 9.5) and a spatula tip of BCIP (Roth, 6368.2) was prepared. The membrane was incubated shaking on a platform at RT in the staining solution until bands became visible (30–45 min). The blot was documented by using a commercial scanner (Cannon; 800 dpi) without densitometry-based correction.

### RNA extraction and quantitative RT-PCR

Sample preparation *: S. aureus* USA300 JE2 from overnight cultures in BHI-T were harvested (1 min at 15871 rcf) and washed once with PBS. Bacteria were adjusted to OD_600_ = 0.05 in 10 ml sTMS (in 100 ml flasks), different concentrations of biotin (0, 1, 4, and 10 nM) were added and the cultures were grown to mid-log phase (~5 h) at 37°C with agitation. The pellet was resuspended in 1 ml Trizol (Invitrogen) and transferred to tubes containing glass beads (MN Bead Tubes Typ B, 740812.50). Cells were disrupted using FastPrep-24 (6.5 m/s for 2x 30 sec) and centrifuged (1 min at 15871 rcf). Supernatants were transferred into fresh Eppendorf tubes and stored at −80°C overnight. RNA isolation and cDNA synthesis: For RNA isolation, the samples were first thawed at RT. After adding 200 μl chloroform, the samples were vortexed for 30 s and incubated for 3 min at RT. After centrifugation at 12 000 × g for 15 min at 4°C (Eppendorf Centrifuge 5415R), 600 μl of the supernatants (aqueous phase) were transferred into new Eppendorf tubes filled with 500 μl isopropanol. The samples were inverted 30 times and incubated for 10 min at RT prior centrifugation at 12 000 × g for 20 min at 4°C (Eppendorf Centrifuge 5415R). The pellet was washed once with 500 μl 70% EtOH and centrifuged at 12 000 × g for 20 min at 4°C (Eppendorf Centrifuge 5415R). After removing the supernatants, the pellets were dried for 1 h under the hood. Residual DNA was removed by DNase treatment using the DNase I, RNase-free of Thermo Scientific (EN0521) and incubation at 37°C for 30 min. After adding 90 μl nuclease-free water (Qiagen, 129114), RNA samples were cleaned up using the NucleoSpin RNA Clean-up Kit (Macherey-Nagel, 740948.50) and RNA concentrations were measured with the nanodrop (FastGene NanoSpec Photometer, FG-NP01, NIPPON Genetics). RNA samples were snap-frozen in liquid nitrogen and stored at −80°C. For cDNA synthesis, 200 ng RNA and the iScript Adv cDNA Kit for RT-qPCR (Bio-Rad, 1 725 038) was used. The cDNA was snap-frozen in liquid nitrogen and stored at −80°C. qPCR: For qPCR, primers for the housekeeping gene *gyrB* and the gene of interest *bioA* were designed using Primer3plus (Suppl. Tab. S2) and mixed to a 25 μM per primer stock in nuclease-free water (Qiagen, 129 114). A master mix with 150 nM primer (end concentration), 2x QuantiNova SYBR Green PCR Kit (Qiagen, 208 054) and water was prepared and 5 μl was transferred to a 96-wel Hard-Shell PCR plates (Bio-Rad, HSP9655). A concentration of 50 ng cDNA in 5 μl total volume was also transferred to the plate and mixed properly. The plate was sealed with Microseal “B” seal (Bio-Rad, MSB1001) and run in a C1000 Touch Thermal Cycler with the CFX96 Touch Real-Time PCR detection system (Bio-Rad) under following conditions: 50°C for 90 sec, 95°C for 10 min, and 39 cycles at 95°C for 15 s, 60°C for 30 sec, and subsequently 95°C for 15 sec and 60°C for 45 sec.

### Membrane fluidity assay

Bacteria from overnight cultures in TSB were harvested (1 min at 15871 rcf) and washed once with PBS. Bacteria were adjusted to OD_600_ = 0.05 in 10 ml sTMS (in 100 ml flasks), different concentrations of biotin were added and the cultures were grown for 24 h at 37°C with agitation. Cells corresponding to 1 ml OD_600_ = 1 were harvested and frozen at −20°C until usage.

To determine the membrane fluidity, the Membrane Fluidity Kit from Abcam (ab189819) was used according to the manufactures protocol. Pellets stored at −20°C were thawed and washed once with 400 μl PBS + 20% sucrose. After centrifugation at 8000 rcf for 5 min, the supernatants were discarded and the pellets were treated with 400 μl 20% sucrose, 0.01% F-127 and 10 μM PDA for 1 h in the dark at RT. The samples were centrifuged as above, washed once with 400 μl PBS + 20% sucrose and taken up in 200 μl PBS + 20% sucrose. Two technical replicates of 100 μl were transferred into a black 96-well microtiter plate (Greiner, 655 097) and the fluorescence was measured by excitation at 360 nm and emission at 405 nm (monomer) and 460 nm (excimer). Higher membrane fluidity correlates with higher excimer to monomer ratio.

### *In silico* analysis

Genome sequence of *S. aureus* USA300 FPR3757 and assembled genomes of species from our collection were screened for annotated core biotin biosynthesis genes *bioD*, *bioA/bioK*, *bioB*, *bioF* encoded either in ORFs or distributed in the genomes, as well as biotin transporter genes *bioY* and *yigM*/*bioP* using Artemis [43]. A BLAST search was performed using reference genes of *S. aureus* USA300 FPR3757 *bioD* (SAUSA300_2373), *bioA* (SAUSA300_2372), *bioB* (SAUSA300_2371), *bioF* (SAUSA300_2370), *bioY* (SAUSA300_2233), and *yigM/bioP* of *P. aeruginosa* PA01 (PA3474) using NCBI BLAST. Additionally, whole genomes where blasted against the reference genes to identify positive hits using NCBI BLAST [44]. All BLAST results are summarized in Suppl. Tab. S3.

Genetic relatedness of nasal commensals used in this work was visualized by similarity of the 16S rRNA loci. The respective sequences were obtained from NCBI Refseq in all cases except for *C. kefirresidentii*. Here the 16S rRNA locus was directly retrieved from the respective WGS data of the used isolate. The obtained sequences were aligned using the Culstal Omega algorithm provided in the msa package [45]. A phylogenetic tree was created from the aligned sequences using the UPGMA method provided in the phangorn package [46].

### Liquid co-cultural growth in sTMS

Bacteria were grown overnight in BHI-T and 12.5 μg/ml tetracycline was added for fluorescent *S. aureus* strains containing the GFP-expressing plasmid pC183-S3. Cells were harvested (1 min at 15871 rcf) and washed once with PBS. S. *aureus* USA300 JE2 and mutant strains were adjusted to an OD_600_ = 0.1 in PBS and commensals were adjusted either to OD_600_ = 0.1 (for 1:1 ratio), 0.001 (for 100:1 ratio; used for *Klebsiella michiganensis* 44, *Proteus mirabilis* 90, *Klebsiella oxytoca* 218, and *Bacillus cereus 272*), 1 (for 1:10 ratio used for *S. aureus* 198) or 10 (for 1:100 ratio used for CoNS except *S. haemolyticus* 147 (for SHae147: 1:1)) in PBS. In triple co-cultures, 1 μl of each commensal suspension together with 1 μl of OD_600_ = 0.1 of the fluorescently labeled *S. aureus* strain was mixed in 1 ml sTMS medium and 500 μl was transferred to a 48-well microplate (Nunc, Thermo Scientific). Growth was monitored by measuring the OD_600_ and fluorescence (Em. 480 nm, Ex. 520 nm and Gain 50) every 15 min for 48 h in a BioTek Synergy H1 Reader (Agilent) at 30°C with orbital shaking. By plotting the fluorescence values against the OD_600_, the fluorescence level at OD_600_ = 0.5 was determined and relative proportion of labeled *S. aureus* strains and unlabeled competitors calculated. To determine the fitness of the GFP-labeled strains, obtained OD values (start OD and determined relative end OD) from the cogrowth were background corrected and used to calculate the relative fitness for the GFP-labeled strain (GLS) against the nasal competitor strain (NCS) as previously described [47].


$$ {Relative\ fitness}_{GLS}=\frac{\left( End\ {OD}_{GLS}\times Start\ {OD}_{NCS}\right)\ }{\left( End\ {OD}_{NCS}\times Start\ {OD}_{GLS}\right).} $$

Fitness improvement against competitor strains of USA300 JE2 wildtype over the JE2 *ΔbioY* mutant was quantified as log10-residual of the relative fitness of WT to the assumed equal fitness.


$$ {Fitness\ improvement}_{WT}=\mathit{\log}10\left(\frac{{Relative\ fitness}_{WT}}{{Relative\ fitness}_{\Delta bioY}}\right) $$

### Co-cultural growth on sTMS agar plates

Biotin-depleted sTMS agar was prepared as described above. Bacteria from BHI-T overnight cultures were collected (1 min at 15871 rcf) and washed once with PBS. For different nasal isolates used as a lawns, OD_600_ was adjusted to 0.05 and streaked on plates using sterile cotton swabs. For *S. aureus* JE2 pC183-S3 and *S. aureus* JE2 Δ*bioY* pC183-S3 grown as spot, OD_600_ was adjusted to 1, a 10-fold dilution series was prepared in PBS and 10 μl were spotted on the lawns. The plates were incubated for 48 h at 37°C. Growth of fluorescently labeled *S. aureus* strains were documented by taking photos of the plates with the Intas UV system (Intas GDS Touch 2, Exposure time: 1500) and the FastGene Blue/Green LED flash light (Nippon Genetics; FG-11) and quantified by determination of the minimal dilution that enable visible growth.

## Results

### Appropriate biotin levels are required for normal *S. aureus* USA300 JE2 physiology

**Figure 2:**
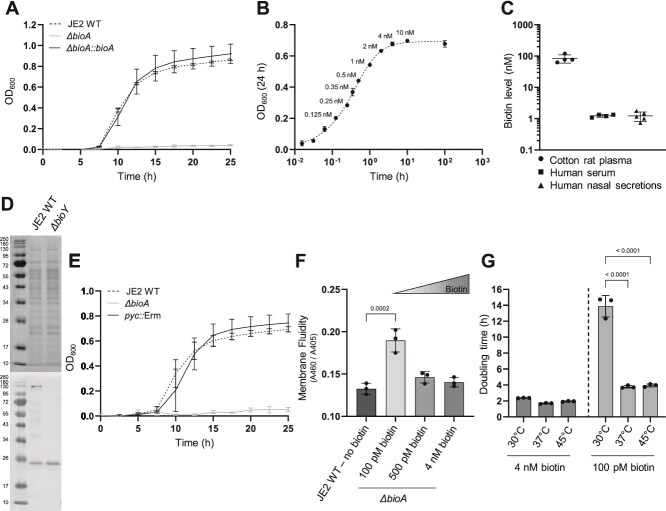
Effects of biotin limitation on *S. aureus* physiology*.* (A) Growth under biotin-limited conditions. Strains were inoculated to an OD_600_ = 0.0001 in 500 μl streptavidin-treated (biotin-free) TMS medium (sTMS) in a 48-well plate and growth was monitored every 15 min for 25 h at 37°C using an Epoch2 orbital reader. For reasons of clarity, only values taken every 2.5 h are displayed. Mean and SD of three independent experiments is shown. (B) Biotin titration. *S. aureus ΔbioA* was inoculated as in (A) and supplemented with different biotin concentrations. Growth was monitored as in (A) and OD_600_ at timepoint 24 h was plotted against the biotin concentration. Mean and SD of three independent experiments are shown. (C) Biotin levels in cotton rat plasma and different human body fluids. Biotin levels were measured using the IDK biotin ELISA kit (K8141) from Immundiagnostik. Mean and SD of four cotton rat plasma samples, four human serum samples and five human nasal secretions are shown. (D) Biotinylated enzymes in *S. aureus*. Streptavidin blot (bottom) and SDS-Page (top) of crude extracts of *S. aureus* USA300 JE2 WT and the pyruvate carboxylase mutant from the Nebraska Transposon Mutant Library (NTML) (*pyc::*Erm*)* grown overnight in TSB. Biotinylated enzymes were detected by using the alkaline phosphatase activity of the streptavidin-AP conjugate. (E) Growth of the Pyc-deficient strain. Growth was monitored as in (A). Mean and SD of three independent experiments are shown. (F) Membrane fluidity. *S. aureus* USA300 JE2 WT and *ΔbioA* were grown in sTMS in flasks with various concentrations of biotin. Membrane fluidity was assessed using the kit from Abcam (ab189819). Mean and SD of three independent experiments are shown. Statistical analysis was performed using ordinary one-way ANOVA. (G) Biotin-dependent growth at different temperatures. The strain *ΔbioA* was grown in sTMS in the presence of either 4 nM or 100 pM biotin at different temperatures and the generation times in exponential phases were calculated. Mean and SD of three experiments are shown statistical analysis was performed using ordinary two-way ANOVA (*P* < .0001).

*S. aureus* USA300 (FPR3757) encodes the *bioDABF* operon (SAUSA300_2373; SAUSA300_2372; SAUSA300_2371; SAUSA300_2370) suggesting that the strain is able to synthesize biotin endogenously from pimeloyl-CoA. Indeed *S. aureus* USA300 JE2 WT (JE2 WT) was able to proliferate in biotin-deficient Tris Minimal Succinate medium (sTMS) (Fig. 2A). This medium was used throughout our experiments as it allows robust, biotin-dependent growth of *S. aureus* as well as of many nasal commensal species. In contrast, a *bioA* deletion mutant (*ΔbioA*) failed to grow under the same conditions and chromosomal reversion (*ΔbioA::bioA*) rescued the phenotype in sTMS (Fig. 2A,). Similar phenotypes were observed in biotin-deficient Synthetic Nasal Medium [18] which mimics the nutritional surrounding within the nasal cavity more closely and is characterized by slower growth rates on lower yields (Suppl. Fig. S1A). In contrast, the *bioA*-deficient strain did not show fitness defects when grown in biotin-rich Tryptic Soy Broth (TSB), speaking against a general fitness disadvantage or secondary site mutations (Suppl. Fig. S1B). We performed titration experiments in sTMS and SNM20 medium to identify the critical biotin concentration needed by *S. aureus*. Biotin enhanced the proliferation of the *ΔbioA* strain in a dose-dependent manner, with 4 nM being sufficient for normal levels of growth akin to the wildtype strain (Fig. 2B and Suppl. Fig. S1A/C). *S. aureus* is known to proliferate within the human host during infection as well as within the human nasal cavity during colonization. We used biotin ELISA assays to investigate if exogenous biotin might be a relevant nutritional source in these habitats. In agreement with previous studies [36], we found human serum to contain ~1 nM biotin (Fig. 2C). Nasal secretions contained very similar concentrations showing that biotin is a scarce nutrient in the nose. However, biotin levels are within the physiologically relevant range for *S. aureus* in both human habitats. As described before, rodent serum contains a much higher concentration of biotin (~100 nM) suggesting that biotin restriction is not relevant in rodent model systems [36].

The effects of biotin limitation on *S. aureus* physiology are not well known. Only two *S. aureus* enzymes are thought to be biotinylated [40, 48]: firstly, the pyruvate carboxylase (Pyc) which enables usage of pyruvate for gluconeogenesis [49] and, secondly, the acetyl-CoA carboxylase (Acc) a crucial enzyme for long-chain fatty acid biosynthesis [50]. *S. aureus* USA300_FPR3757 encodes Pyc (SAUSA300_1014) and two alleles of Acc (SAUSA300_1476 and SAUSA300_1564). Streptavidin blots of JE2 WT whole cell extracts showed two distinct bands, corresponding to Pyc (128.5 kDa) and Acc (17 kDa). The Acc band appears at ~25 kDa rather than predicted 17 kDa, a phenomenon that has been reported previously [51] (Fig. 2D). However, two bands indicating expression of both Acc proteins (16.8 kDa and 17.12 kDa) could not be identified. A *pyc*-deficient mutant (*pyc::*Erm*,* derived from the Nebraska Transposon Mutant Library (NTML) [52]) showed absence of the 128.5 kDa band in streptavidin blots (Fig. 2D) but did not show a growth deficit under biotin-limiting conditions (Fig. 2E). This suggests that the enzyme is dispensable under the experimental conditions used. Mutants with defects in any of the two *acc* genes were neither available in the NTML nor in more dense transposon mutant libraries [53], suggesting essentiality. This is in line with previous reports showing that inactivation of *acc* results in fatty acid auxotrophy [54]. Accordingly, we hypothesized that biotin limitation interferes with fatty acid biosynthesis in the *ΔbioA* mutant. Indeed, compared to the wildtype, strict biotin limitation (100 pM) resulted in a strong increase of membrane fluidity in the *ΔbioA* mutant. Increased biotin supplementation rescued the phenotype (Fig. 2F). Appropriate membrane fluidity is needed for adaptation to different temperatures [55–57]. Calculation of generation times of the *ΔbioA* mutant showed that biotin limitation heavily impacted fitness, especially at a growth temperature of 30°C (Fig. 2G).

### Biotin transporter BioY improves the fitness of *S. aureus* and reduces biotin loss to the environment

**Figure 3:**
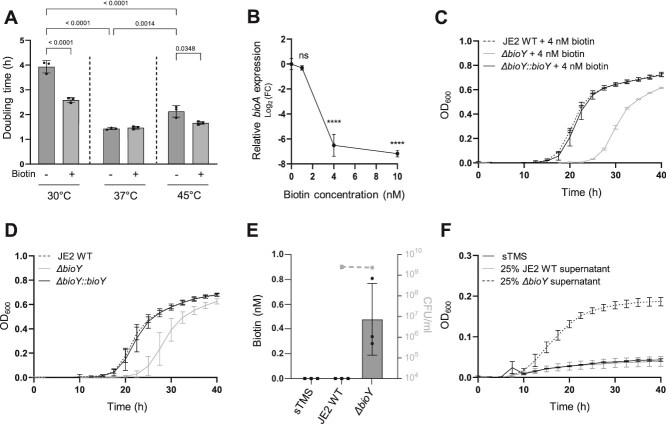
Effects of exogenous biotin. (A) *S. aureus* USA300 JE2 WT profits from exogenous biotin. *S. aureus* was grown in presence (+) or absence (−) of 4 nM biotin at various temperatures in 48 well plates and growth was monitored using an Epoch2 plate reader. Growth rates in exponential phase were calculated. Mean and SD of three independent experiments are shown. Two-way ANNOVA was used for statistical analysis (*P* < .0001). (B) Downregulation of biotin biosynthesis under physiological levels of biotin. *S. aureus* JE2 WT was grown to mid-log phase in 10 ml sTMS with indicated biotin levels and cells were harvested. The RNA was extracted and transcribed to cDNA. The expressions level of Δ*bioA* was determined by qPCR and normalized to the expression levels of the housekeeping gene *gyrB*. (C, D) Effects of BioY-dependent biotin acquisition. Strains were inoculated in 48 well format to an OD_600_ = 0.0001 in 500 μl in sTMS medium with (C) or without (D) supplementation with 4 nM biotin. Growth was monitored every 15 min for 40 h at 30°C using an Epoch2 orbital reader. For reasons of clarity, only values taken every 2.5 h are displayed. Mean and SD of three independent experiments are shown. (E) Biotin levels in sTMS and culture supernatants. Biotin levels and CFU/ml in sTMS and of strains grown for 24 h in 10 ml sTMS were assessed using the IDK biotin ELISA kit (K8141) from Immundiagnostik. Mean and SD of three independent experiments are shown for JE2 WT and Δ*bioY*. Filled circles indicate measurable biotin levels in the supernatants. Filled black squares indicate samples in which no quantifiable biotin could be measured. (F) Growth support of *ΔbioA* by *ΔbioY* supernatants. *ΔbioA* was inoculated to an OD_600_ = 0.0001 in 500 μl sTMS containing 25% sterile filtered *S. aureus* USA300 JE2 WT and *ΔbioY* supernatants and growth was monitored in 48-well plates for 40 h at 37°C using an Epoch2 orbital reader. For reasons of clarity, only values taken every 2.5 h are displayed. Mean and SD of three independent experiments are shown.

Between 19 and 20 molecules of ATP are necessary for the production of a single molecule of biotin, making its biosynthesis extremely cost-intensive. We speculated that biotin import might improve the fitness of *S. aureus* by sparing cellular resources. At 30°C, supplementation of biotin deficient medium with 4 nM biotin drastically increased the growth of the prototrophic JE2 WT strain (2,5 h compared to 3,9 h per generation) (Fig. 3A). In contrast, growth-stimulatory effects of exogenous biotin were less pronounced at 45°C and absent at 37°C (Fig. 3A). This reinforces the notion of biotin biosynthesis/acquisition being particularly important at low temperatures. Furthermore, increasing concentrations of biotin (up to 4 nM) resulted in decreased expression of the biotin biosynthesis gene *bioA* (Fig. 3B). This is in line with previous results [58] and suggest that *S. aureus* reduces expression of the costly biosynthesis pathway when internal concentrations are sufficient. *S. aureus* encodes the biotin specific ECF-type transporter BioY for which the hydrolysis of two molecules ATP is needed to import one molecule of the coenzyme. An isogenic deletion mutant of *bioY* (*ΔbioY*) was used to test the contribution of the ECF-type transporter. The *ΔbioY* strain did not show abnormal phenotypes when grown with excess biotin (Suppl. Fig. S2A/B) but an increased lag time along with a reduced final optical density was apparent when grown in presence of 4 nm biotin which is restrictive but sufficient to allow WT levels of growth of the biotin auxotrophic *ΔbioA* mutant (Fig. 3C). These phenotypes were abrogated by chromosomal reversion of the mutation (*ΔbioY::bioY*). In contrast to our initial hypothesis, we observed that the *ΔbioY* mutant displayed an abnormal growth regardless of the presence of biotin (Fig. 3C and D). This phenotype is best explained by the assumption that endogenously synthesized biotin is released and subsequently recaptured by BioY. To further investigate this, we measured the biotin concentration in spent culture supernatants of JE2 WT and *ΔbioY* strains grown without exogenous biotin. Extracellular biotin was not detectable in the supernatants of the WT strain, while those of the *ΔbioY* mutant contained ~500 pM (Fig. 3E). Both strains reached comparable CFU counts at the end of the experiment (Fig. 3E) and did not differ in their autolysis profiles (Suppl. Fig. S2E) suggesting that differences were caused by BioY-dependent regain of biotin and not by different levels of cell death. Using spent culture supernatants of JE2 WT and mutant, we found that only the *bioY* deficient (*ΔbioY*) strain’s supernatant fostered growth of the biotin auxotroph (*ΔbioA*) (Fig. 3F). All phenotypes of *bioY* deficiency were also observed in SNM20 (Suppl. Fig. S2C, D, F, G). Taken together, these data suggest that BioY plays a dual role for the fitness of *S. aureus*. Firstly, it allows acquisition of biotin derived from external sources and secondly it minimizes the loss of endogenously produced biotin. This improves the fitness of the producer but also prevents its usage by other bacteria.

### Nasal commensals show diverse biotin auxotrophies and overproduction phenotypes.

We speculated that release and BioY-dependent uptake of biotin might foster interactions between *S. aureus* and nasal commensals because of biotin limitation in the nose. To investigate this, we used a strain collection of human nasal bacteria isolated from healthy human volunteers [59].

**Figure 4:**
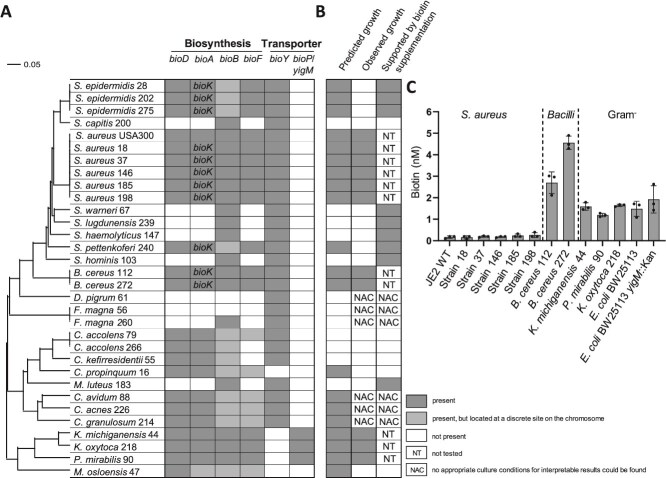
Biotin-dependency of nasal isolates. (A) *In silico* analysis of biotin biosynthesis and transporter genes. Genomes of nasal isolates were grouped by the phylogeny of their 16S rRNA gene and analysed for core biotin biosynthesis genes *bioD, bioA*, *bioB,* and *bioF,* and biotin transporter genes *bioY* and *yigM/bioP.* Dark gray boxes indicate the presence of the gene in an operon, light gray boxes indicate the presence of the gene located at a discrete site on the chromosome and white boxes indicate the lack of the gene. Some strains encode *bioK* instead of *bioA*, catalyzing the same reaction with the difference that BioK uses lysine as amino donor instead of SAM. (B) Predicted and observed growth in sTMS with and without biotin. Predicted growth was assumed for strains containing all four core genes *bioD, bioA*, *bioB,* and *bioF* in their genomes. An OD_600_ > 0.2 after 30 h was regarded as growth. Support by biotin supplementation was regarded as positive when fold change of strains grown in the presence of 10 nM biotin compared to 0 nM biotin was >1. Filled gray boxes indicate growth and no growth is indicated as white boxes. NT: Not tested; NAC: No appropriate culture conditions for interpretable results could be found. (C) Biotin levels in the supernatant of nasal isolates. Strains were grown for 24 h in 10 ml sTMS and biotin levels in supernatants were determined.

We chose representative strains and investigated their genome sequences for the presence of genes for biotin biosynthesis and uptake (Fig. 4A). We assigned bacteria as prototroph when possessing all four core genes *bioD*, *bioA*, *bioB,* and *bioF,* and found putative biotin auxotrophy to be a frequent trait. Among the CoNS, *S. hominis*, *S. lugdunensis, S. warneri, S. capitis,* and *S. haemolyticus* possessed incomplete operons for biotin biosynthesis. The same was true for *Dolosigranulum pigrum*, *Finegoldia magna*, *Micrococcus luteus, Corynebacterium accolens,* and *C. kefirresidentii*. Genes encoding putative biotin acquisition systems were identified in all strains with the exception of *C. propinquum* and *Moraxella osloensis*. However, biotinylated enzymes were detected in representative strains of all species (Suppl. Fig. S3A, B and C) suggesting that auxotrophic strains depend on exogenously available biotin. Biotinylated proteins were also detected in prototrophic strains grown in the absence of exogenous biotin, verifying the validity of our detection method and supporting the idea that biotin is endogenously synthesized (Suppl. Fig. S3D).

We tested the ability of representative strains to proliferate in biotin-deficient medium. None of the strains predicted to be biotin auxotrophs were able to grow under these conditions (Fig. 4B and Suppl. Fig. S4A). However, several strains predicted to be prototrophic for biotin also failed to grow (including *S. epidermidis*, *S. pettenkoferi, M. osloensis,* and all tested *Corynebacteria*). In agreement with previous reports [60], addition of extracellular biotin improved the growth of CoNS as well as of *M. luteus* up to 5-fold. However, exogenous biotin did not rescue the growth of *Corynebacteria* (Fig. 4B and Suppl. Fig. S4B). This suggests that under our experimental conditions biotin limitation was growth-limiting for several isolates, while additional limitations existed for others.

All strains that were capable of proliferation under biotin-free conditions reached final cell densities between 1 × 10^8^ and 5 × 10^9^ CFU / ml (Suppl. Fig. S4C). Spent medium of all strains contained biotin but the amounts varied considerably (Fig. 4C). All *S. aureus* strains released low amounts of biotin (~200 pM) while two *B. cereus* strains produced 15 to 25-fold more. All nasal Gram-negative species released 6–8-fold more biotin than *S. aureus*. We included the laboratory strain *E. coli* BW25113 and the strain *yigM::*Kan, lacking the biotin importer. Supernatants of BW25113 contained similar amounts of biotin compared to those of other Gram-negative species. A trend towards increased biotin accumulation in the *yigM* mutant (*yigM::*Kan) (Fig. 4C) was observed, suggesting that the principle of biotin loss and regain might be conserved across species.

Altogether these datasets indicate that nasal bacteria show considerable differences within their need of exogenous biotin as well as within their capacity to release biotin to the environment.

### Release of biotin and BioY-dependent uptake promotes interaction of nasal bacteria with *S. aureus*

**Figure 5:**
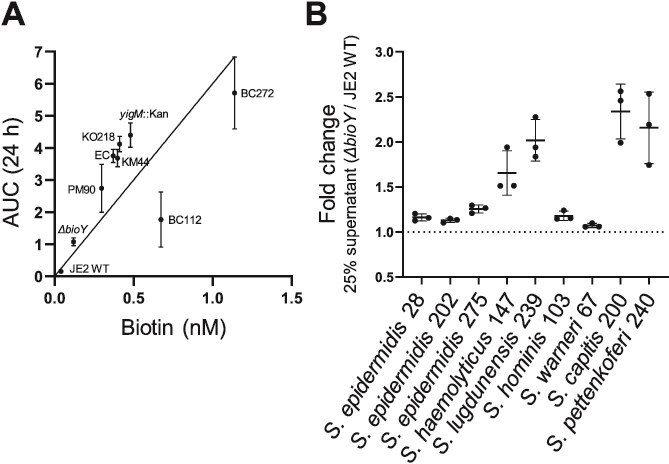
Biotin-containing supernatants support *Staphylococci* growth in sTMS. (A) Growth support of *S. aureus ΔbioA* by supernatants of nasal commensals*.* The strain *ΔbioA* was inoculated to an OD_600_ = 0.0001 in 500 μl sTMS containing 25% sterile filtered *S. aureus* JE2 WT, *ΔbioY,* and commensal supernatants (EC: *E. coli* BW25113*;*  *yigM::*Kan: *E. coli* BW25113 *yigM::*Kan*;*  BC112*: B. cereus* 112*;*  BC272*: B. cereus* 272*;*  KM44*: K. michiganensis* 44*;*  PM90*: P. mirabilis* 90 and KO218*: K. oxytoca* 218*).* Growth was monitored for 24 h at 37°C in 48-well plates using an Epoch2 orbital reader. Area under the curve was determined after 24 h. Shown are the mean and SD of three independent biological replicates. The relationship between biotin concentration in the supernatant and the growth increase of *ΔbioA* was assessed by computing Pearson correlation coefficient (*r* = 0.73 and *P* = .0250). (B) Growth of CoNS strains in sTMS with 25% supernatants of *S. aureus* JE2 WT and *ΔbioY*. CoNS strains (SW67: *S. warneri* 67; SHo103: *S. hominis* 103; SHae147: *S. haemolyticus* 147; SC200: *S. capitis* 200; SL239: *S. lugdunensis* 239; SP240: *S. pettenkoferi* 240; SE28: *S. epidermidis* 28; SE202: *S. epidermidis* 202, and SE275: *S. epidermidis* 275) were inoculated to an OD_600_ = 0.0001 in 500 μl sTMS containing 25% *S. aureus* JE2 WT or 25% *ΔbioY* supernatant for 24 h at 37°C. Fold change was determined at OD_600_ = 24 h_._ The mean and SD of three independent biological replicates are shown.

We used dilute culture supernatants to assess if the different biotin levels produced by nasal commensals increased the proliferation of *S. aureus*. Indeed, the biotin auxotrophic *S. aureus* mutant (*ΔbioA*) profited from culture supernatants of commensals and the degree of growth promotion was directly correlated (*r* = 0.73; *P* = .0250) to the amount of biotin present in the supernatants (Fig. 5A). Similarly, solid-phase co-culture assays showed that commensals stimulated *S. aureus* growth in a biotin-dependent manner and to very different extents (Suppl. Fig. S5). These data strongly suggest that different levels of biotin are excreted during growth of nasal commensals and that this biotin is accessible to *S. aureus*. In the reciprocal experiments, the dilute culture supernatant of *S. aureus* WT was a poor biotin source for nasal auxotrophic CoNS, while that of the *ΔbioY* mutant substantially increased the growth of several strains including *S. capitis*, *S. lugdunensis*, *S. haemolyticus,* and *S. pettenkoferi* (Fig. 5B), showing that the strains are able to acquire biotin released by *S. aureus*.

**Figure 6:**
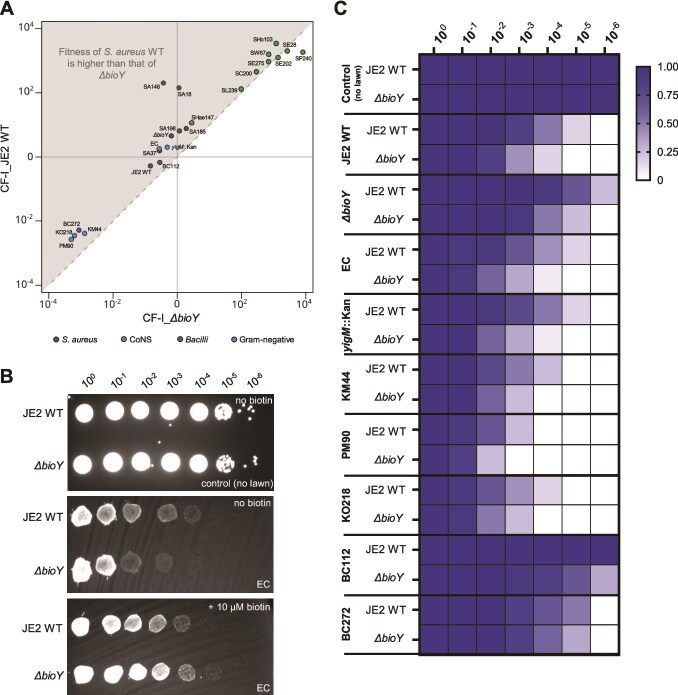
Co-cultivation of JE2 WT and *ΔbioY* with commensals under biotin-limited conditions. Unlabeled bacterial competitor strains were grown with either GFP- labeled *S. aureus* JE2 WT (JE2 WT pC183-S3) or GFP-labeled *S. aureus* JE2 *ΔbioY* (*ΔbioY* pC183-S3). (A) For both *S. aureus* strains the competitive fitness index (CF-I) against each competitor was calculated and put in proportion. JE2: *S. aureus* USA300 JE2; *ΔbioY*: *S. aureus* USA300 JE2 *ΔbioY;*  SA18: *S. aureus* 18; SA37: *S. aureus* 37*;*  SA146: *S. aureus* 146; SA185: *S. aureus* 185; SA198: *S. aureus* 198; EC: *E. coli* BW25113*;*  *yigM::*Kan: *E. coli* BW25113 *yigM::*Kan*;*  BC112*: B. cereus* 112*;*  BC272*: B. cereus* 272*;*  KM44*: K. michiganensis* 44*;*  PM90*: P. mirabilis* 90, and *KO218: K. oxytoca* 218*;*  SW67: *S. warneri* 67; SHo103: *S. hominis* 103; SHae147: *S. haemolyticus* 147; SC200: *S. capitis* 200; SL239: *S. lugdunensis* 239; SP240: *S. pettenkoferi* 240; SE28: *S. epidermidis* 28; SE202: *S. epidermidis* 202, and SE275: *S. epidermidis* 275. The dashed line represents the assumed equal fitness of JE2 WT and JE2 Δ*bioY.* Dots represent mean results of three independent biological replicates for each group (detailed results can be obtained from Suppl. Fig. 7). (B, C) Competition of *S. aureus* JE2 WT and *ΔbioY* against commensals on biotin-deficient sTMS agar. Unlabeled prototrophic bacterial competitor strains were grown as a confluent lawn on sTMS agar plates. GFP-labeled *S. aureus* JE2 WT pC183-S3:GFP and *S. aureus* JE2 *ΔbioY* pC183-S3:GFP were doted in 10-fold dilutions on top of the lawns and dilutions forming spots were quantified. (B) Example plates. Agar plate-based competition of WT and *ΔbioY* mutants against lawns of *E. coli BW25113* in presence or absence of 10 μM biotin. (C) Quantification of co-cultivation on sTMS plates. Growth of 10-fold diluted GFP-labeled *S. aureus* stains on the lawns of diverse prototrophic competitors was quantified as follows: 1: Full bright spot or clear individual colonies; 0.75: bright rims of the spots with darker inner areas; 0.5: spot is weak but clearly visible; 0.25: spot is barely visible. Indicated is the mean of three biological replicates. EC: *E. coli* BW25113*;*  *yigM::*Kan: *E. coli* BW25113 *yigM::*Kan*;*  KM44*: K. michiganensis* 44*;*  PM90*: P. mirabilis* 90*;*  KO218*: K. oxytoca* 218; BC112*: B. cereus* 112, and BC272*: B. cereus* 272.

Together our experiments support the hypothesis of biotin-sharing within the nasal microbiome. Biotin is released by *S. aureus* as well as by other prototrophic commensals to form a generally accessible pool. All strains, prototrophic as well as auxotrophic can access this pool, to optimize growth and competitive fitness. However, the relevance of biotin acquisition for *S. aureus* in the context of endogenous biosynthesis and competing microbes remains unclear. To investigate this, we performed competitive fitness analysis to elaborate the fitness advantage associated with expression of BioY for the prototrophic *S. aureus* strain when competing with diverse commensals. *S. aureus* JE2 WT and the isogenic *ΔbioY* mutant were fluorescently labeled by introduction of the GFP-encoding plasmid pC183-S3. Labeled strains showed a fluorescent signal in linear proportion to the optical density (Suppl. Fig. S6). Both labeled strains were grown independently under biotin-limited conditions in the presence of unlabeled competitors. In all cultures a constant increase in OD was observed while GFP signal was impacted differently by the various competitor species. Accordingly, GFP/OD correlation allows to assess the growth of the *S. aureus* strain as well as that of the competitor in each culture. We used OD/CFU correlation to assess the proportion of strains at an OD600 = 0.5 and competitive fitness indexes (CF-I) [47] for the WT and the *bioY*-deficient strains against all competitors were calculated and set in proportion (Fig. 6A)

In general, *S. aureus* JE2 exhibited varying degrees of fitness when competing against other bacterial species. Both strains (WT and *ΔbioY*) were rapidly outcompeted by *K. michiganensis*, *P. mirabilis*, *K. oxytoca*, and *B. cereus* 272 (CF-Is of WT and BioY against the commensals around 10^−3^), but maintained relatively equal fitness when co-cultured with *E. coli*, *B. cereus* 112, *S. haemolyticus*, and unrelated *S. aureus* strains (CF-Is in the range of 10^0^). In contrast, both *S. aureus* strains displaced *S. lugdunensis*, *S. capitis*, *S. epidermidis*, *S. warneri*, *S. pettenkoferi*, and *S. hominis* (CF-Is in the range of 10^3^). This general fitness was not correlated to biotin prototrophy or auxotrophy of the competitors. This is also not expected as the molecular reasons determining competitive fitness are multilayered and competition for other nutrients as well as general differences in generation times or even direct interference do also contribute [61]. However, with the exception of *S. epidermidis* 202, *S. epidermidis* 28, and *S. pettenkoferi*, the fitness index of *S. aureus* WT was always higher than that of the *bioY*-deficient strain when tested against the same competitors (gray area of Fig. 6A and Suppl. Fig. S7), highlighting the general importance of biotin acquisition for *S. aureus*. The magnitude of this effect was strain-dependent, with particularly strong differences observed for *S. aureus* strains SA146 and SA18, which performed significantly better in co-culture with the *ΔbioY* mutant: This strongly suggest that the strains profit from increased biotin loss in the BioY deficient strain (Suppl. Fig. S7D). We used the *bioA*-deficient strain in similar competition experiments, to investigate if biotin secreted by commensal does actively contribute to the competitive interactions. Indeed, we found prototrophic commensals to support growth of the auxotrophic strain, showing that *S. aureus* can acquire biotin released by commensals also under direct competition (Suppl. Fig. S8). Finally, we investigated the importance of BioY in multispecies communities (Suppl. Fig. S9). The relevance of BioY was particularly evident when competing with two strains simultaneously that show a general superior fitness against *S. aureus* (*K. michiganensis* 44 and *B. cereus* 272). Similarly, BioY expression was relevant in competition against two species of comparable fitness to *S. aureus* (*E. coli*, *B. cereus* 112) but of little relevance against two combined species with inferior fitness (*S. warneri* 67, *S. lugdunensis* 239). These data suggest that especially under conditions of severe competition, the improved acquisition / reduced loss of biotin improves the fitness of *S. aureus* substantially.

Increased fitness of the WT compared to the BioY deficient strain was also apparent when bacteria were grown on sTMS agar surfaces, which mimic adherent growth within the nasal cavity more closely. When competing with confluent lawns of commensal competitor bacteria, the BioY-deficient strain needed an inoculum density increased by one to three orders of magnitude to form visible colonies (Fig. 6B and C). This demonstrates that competition for biotin is also relevant during surface attached, biofilm-like growth closely resembling the conditions within the nasal cavity.

## Discussion

Biotin is an essential trace nutrient. Bacteria need low nanomolar concentration of biotin for normal growth. Indeed, 10 nM is needed by *P. aeruginosa* [62] and 10 nM was reported to increase growth rates of *S. aureus* [40]. We found *S. aureus* to require ~4 nM biotin under our experimental conditions. Below this threshold *S. aureus* showed reduced growth yields, especially at 30°C which mimics the conditions within the nasal cavity where the bacterium is a frequent commensal [63]. While *S. aureus* contains two biotinylated enzymes we identified the acetyl-coA carboxylase (Acc) as the major driver of this phenotype. Acc is important for fatty acid biosynthesis and we found biotin limitation to increase membrane fluidity, suggesting disturbance of membrane integrity which is in line with previous reports [64–66]. Additionally, it was shown that biotin limitation reduced fibrinolytic activity along with production of phosphatase and coagulase and resulted in increased susceptibility to antimicrobials, highlighting the impact of appropriate biotin levels for *S. aureus* physiology and virulence [67]. Pyruvate carboxylase (Pyc), the second biotin-dependent enzyme of *S. aureus* catalyzes the carboxylation of pyruvate to gain oxaloacetate. This is needed to replenish the TCA cycle for gluconeogenesis [68]. However, the C4 carbohydrate succinate is the dominant carbon sources in nasal secretions [18] and within sTMS used herein. It is therefore not surprising that the pyruvate carboxylase was dispensable under our experimental growth conditions.

The interplay between endogenous biosynthesis and BioY-dependent acquisition of exogenous biotin remains poorly understood. However, it is well known that endogenous biosynthesis is controlled by BirA and transcriptionally repressed in the presence of biotin [40, 58]. Most likely this allows the bacterium to save ATP and channel it in other biosynthetic processes.

Our experiments showed BioY to confer a distinct fitness benefit for *S. aureus* with and without exogenous supplementation of biotin. Additionally, *bioY* deficient strains accumulated more biotin in the supernatant than the wildtype strain, strongly suggesting that *S. aureus* constantly loses endogenously synthesized biotin to the environment and that this problem is minimized by BioY-dependent reacquisition. Similar results were shown for genetically engineered *E. coli* producing vitamin B_12_ (cobalamin) [69]. In that study, the cobalamin transporter mutant *ΔbtuF* accumulated much more vitamin B_12_ in the supernatant than the wildtype [69]. The precise mechanism of excretion of central metabolites is not well investigated. However, the most accepted theory is that metabolites are part of the exometabolome due to metabolic overflow [70, 71]. This is mainly described for major fermentation products (e.g. lactate, acetate, and ethanol) but newer assumptions also include secretion of primary metabolites (e.g. amino acids and nucleosides) [71–73]. A strain-specific secretion of isoleucine and other organic acids due to overflow metabolism during growth in eukaryotic cell medium RPMI has also been described for *S. aureus* [74]. Nonetheless, metabolomic data indicate that all secreted intracellular metabolites cannot solely rely and be explained by intracellular metabolic overflow [75]. Other molecular mechanisms resulting in externalization of metabolites could include transport via facilitated diffusion through nonspecific mechanosensitive channels with pore sizes of ≤2.8 nm [76], loss of membrane integrity, and cell lysis [77]. Using *E. coli*, it was shown that uptake of biotin is energy-dependent and works against the concentration gradient while its release is energy-independent and follows the concentration gradient, suggesting a diffusion-like mechanism [38]. Most likely secretion of biotin by *S. aureus* and other prototrophic nasal bacteria is caused by similar mechanisms. Accordingly, central metabolites can be regarded as public goods that can impact the physiology of other species and foster collaborative and competitive activities within bacterial communities. In the context of the nasal microbiome, it has been shown that classically intracellular coproporphyrins are found in culture supernatants of cutibacteria and induce aggregation of *S. aureus* [78]. We found extensive variation in biotin levels released by nasal commensals. Exogenous biotin stimulated biotin-dependent growth of *S. aureus* in a concentration-dependent manner in both cell-free and co-culture experiments. This demonstrated that *S. aureus* profits from biotin released by nasal communities. Similar vitamin-dependent interactions were predicted and demonstrated to shape dynamics within human gut microbiomes [79–86] and, also within marine microbial communities [87].

Vitamin auxotrophies have been shown to drive co-dependency and cross-feeding within different bacterial communities [85, 88, 89]. Here we found biotin auxotrophy to be a common trait in CoNS and in *D. pigrum*. This has also been observed by others [90, 91] and our *in silico* screening suggests that also *F. magna* relies on biotin produced by others. However, experimental evidence to support this is currently lacking. Human nasal secretions contain only ~1 nM biotin. The origin of biotin in nasal secretions remains unclear. It seems possible that it derives from metabolic overflow from nasal commensals, but is equally likely that it derives directly from human cells producing nasal secretions such as goblet cells (which release mucins) [92], glandulae nasales (producing seromucous fluid) [93] or ductus nasolacrimalis (tear fluid) [94] or even from disintegrated epithelial cells [95]. However, despite being available, the biotin concentration is at the low end of the physiologically needed concentration and competition for the cofactor by auxotrophs is most likely fierce. Here it has to be considered that besides biotin, biosynthesis-intermediates might be released and acquired. This might be a relevant source of biotin for species with fragmented biosynthesis pathways as observed for several nasal commensal species. BioY-dependent acquisition of biotin appears to play a dual role for *S. aureus*. Firstly, it allows regain of endogenously synthesized biotin that might otherwise be lost to the environment. Secondly, it effectively reduces the amount of exogenous biotin to minimize the support of competing auxotrophs within the same habitat. In line with this we found that biotin containing culture supernatants of the BioY-deficient strain fostered growth of auxotrophic CoNS, while those of the wildtype did not. Similarly, competitive fitness of auxotrophic commensals was increased when co-cultured with *ΔbioY* strain. Similar observations were made for the cobalamin transporter BtuF mutant in *E. coli* providing more cobalamin during co-culture and making *ΔbtuF* a better mutualistic partner [69]. Strains SA18 and SA146 profited strongly from competition with the *bioY* deficient strain that provides increased levels of biotin. These strains were reported before to be auxotrophic for the amino acid tyrosine and to be adapted to specific bacterial communities providing this amino acid [59]. It seems possible that these strains carry multiple metabolic constrains including reduced biotin biosynthesis that makes them dependent on special producer communities

The concept of metabolic overflow and reacquisition of the publicly available goods might be of general relevance, especially within a nutrient-limited environment such as the nasal cavity. Auxotrophic “cheaters” that rely on the metabolic capacity of other bacteria are frequently observed [87, 96–98]. In the nasal microbiome the sharing of siderophores to satisfy iron requirement is similar to the sharing of biotin. Several nasal commensals do not produce siderophores and are reliant on those produced by others fostering their own fitness and decreasing that of the producer [20].

The acquisition of costly metabolic molecules and the associated downregulation or even inactivation of endogenous metabolic pathways represents a common bacterial strategy to optimize growth. We demonstrate that well defined biotin transporters (*bioY/ yigM* [34, 99]) are encoded by prototrophic and auxotrophic strains alike, suggesting a general ability to import extracellular biotin. This appears ecologically worthwhile as acquisition allows sparing of ~18 molecules ATP that can be reallocated towards other cellular processes such as biomass production and replication. Accordingly, stringent transcriptional control of biotin biosynthesis by internal levels is demonstrated for many prototrophic phyla present in the nasal microbiome including *Firmicutes* (*Staphylococcus* spp, *Bacillus cereus*), *Actinobacteria* (corynebacteria spp), and *Proteobacteria* (*E. coli, P. mirabilis, and M. osloensis, Klebsiella* spp [40, 100–104]. Additional, computational analysis regarding conservation of biotin biosynthesis regulators and their DNA-binding motifs suggest tight regulation to be a conserved phenomenon within eubacteria [105].

Specialization of bacteria to synthesize or scavenge biotin might not be due to energetic costs but might also reflect the ecological niche and associated optimal growth rates. Slow growing bacteria may conserve energy by minimizing biosynthesis, while faster growers like *S. aureus* may exploit exogenous nutrients for rapid replication when available. We found most coagulase negative staphylococci to be auxotrophic for biotin suggesting that they are adapted to environmental conditions that supply appropriate amounts of biotin for their dedicated niche and replication rate. In contrast, *S. aureus* produces biotin and appears keen not to lose and share the metabolite with the nasal community. This might indicate that for *S. aureus* the external concentrations are frequently insufficient for its lifestyle which is characterized by strong proliferation and rapid consumption of external resources such as carbohydrates [106].

## Conclusion

In conclusion, our study demonstrates that, despite endogenous synthesis, the acquisition of exogenous biotin is crucial for optimal fitness of *S. aureus*. This is of particular importance in the context of auxotrophic competitors that acquire biotin synthesized and released by other organisms.

## Supplementary Material

Suppl_Figures_Changed_wraf248

3_Supplementary_Tables_wraf248

Supplementary_Methods

## Data Availability

Sequence data that support the findings of this study have been deposited as BioProject to NCBI with the primary accession numbers PRJNA1199961 and PRJNA1247435. Other data are provided within the manuscript or supplementary information files.

## Fundings

We acknowledge funding by the German Center of Infection Research “DZIF” TTU 08.708_00 to S.H. Additionally, we acknowledge infrastructural funding by the Deutsche Forschungsgemeinschaft “DFG” (German Research Foundation) in the frame of Germany’s Excellence Strategy—EXC 2124-390 838 134/EXC-2124/1-05.002_0 (S.H.). We acknowledge support from the Open Access Publication Fund of the LMU.
