# Supplementary material for: Competitive fitness of Staphylococcus aureus against nasal commensals depends on biotin biosynthesis and acquisition
Source: ISME J. 2025 Nov 4;19(1):wraf248. doi: 10.1093/ismejo/wraf248 (PMC12642757; doi:10.1093/ismejo/wraf248)
Supplement: Suppl_Figures_Changed_wraf248 [file suppl_figures_changed_wraf248.pdf]

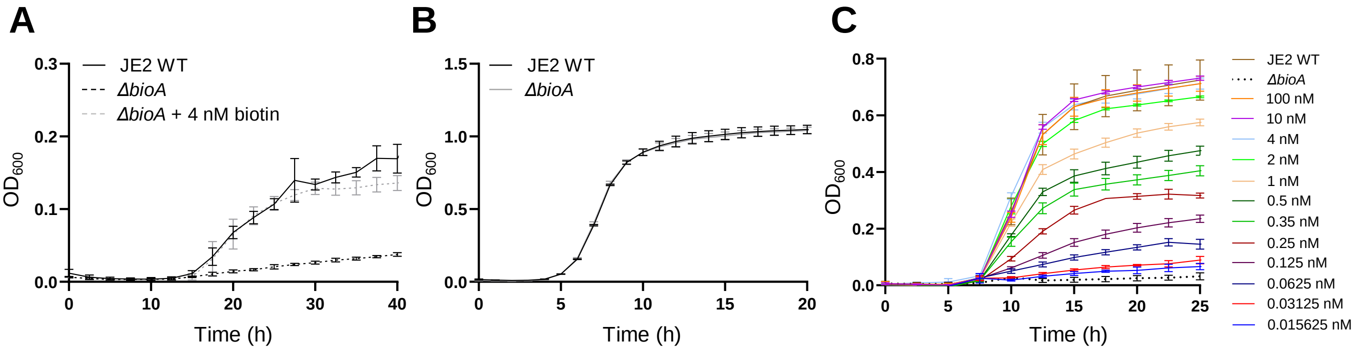

Supplementary Figure 1: Control experiments for *S. aureus ΔbioA* (A) *bioA* dependency of *S. aureus* in SNM20. *S. aureus* JE2 and *ΔbioA* were inoculated to an OD_{600} = 0.0001 in 500 µl biotin-deficient 20x Synthetic Nasal Medium (SNM20) (see Suppl. Methods) in a 48-well plate. Biotin was supplemented as indicated. Growth was monitored every 15 min for 40 h at 37°C using an Epoch2 orbital reader. For reasons of clarity, only values taken every 2 h are displayed. Mean and SD of three independent experiments are shown. (B) Growth of *S. aureus* JE2 and *ΔbioA* in TSB. *S. aureus* JE2 and *ΔbioA* were inoculated to an OD_{600} = 0.0001 in 500 µl Tryptic Soy Broth (TSB) in a 48-well plate. Growth was monitored every 15 min for 20 h at 37°C using an Epoch2 orbital reader. For reasons of clarity, only values taken every 1 h are displayed. Mean and SD of three independent experiments are shown. (C) Effect of biotin titration on *S. aureus ΔbioA* growth. *S. aureus ΔbioA* was inoculated to an OD_{600} = 0.0001 in 500 µl streptavidin-treated (biotin-free) TMS medium (sTMS) in a 48-well plate and supplemented with biotin as indicated. Growth was monitored every 15 min for 25 h at 37°C using an Epoch2 orbital reader. For reasons of clarity, only values taken every 2 h are displayed. Mean and SD of three independent experiments are shown.

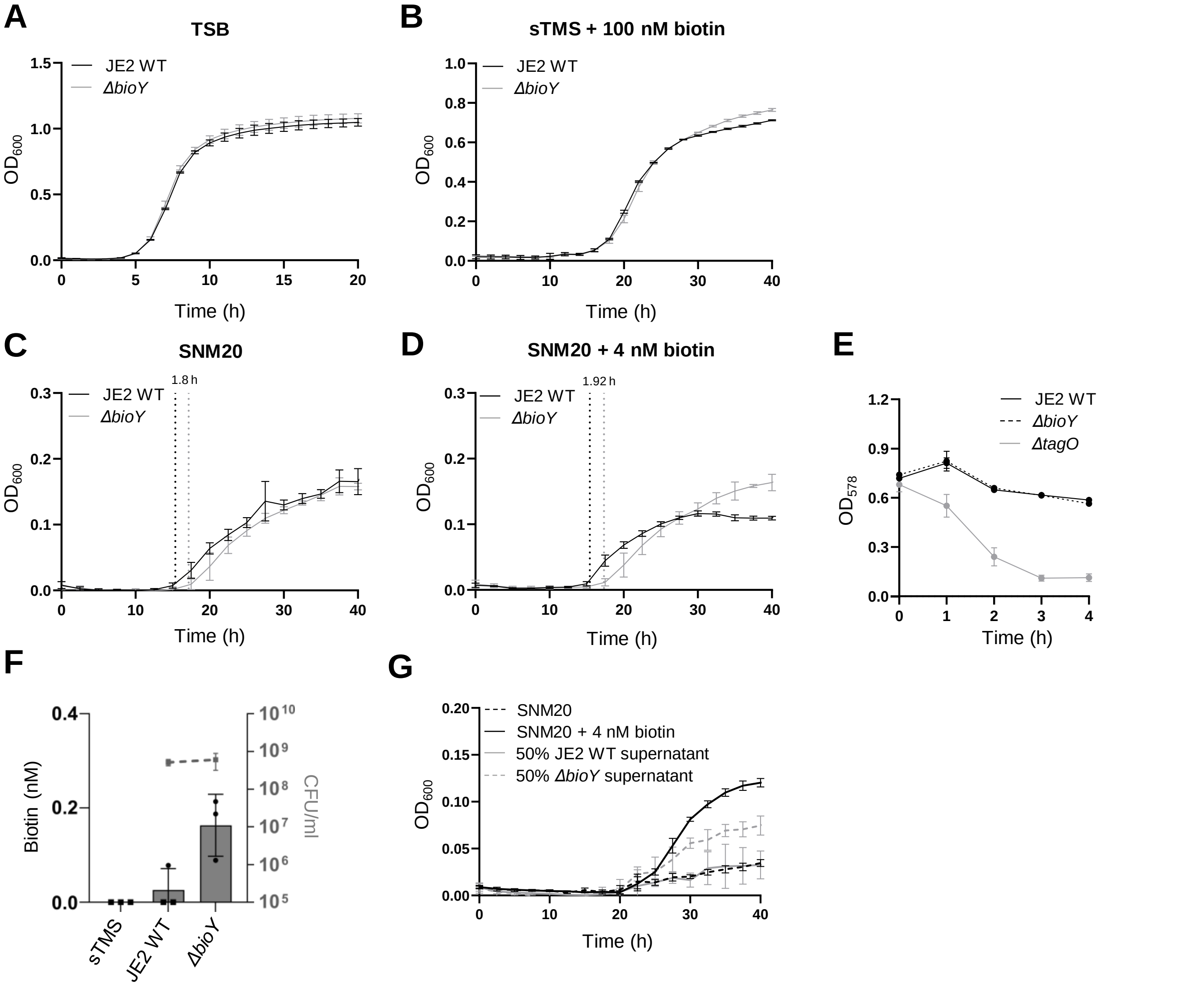

Supplementary Figure 2: Control experiments for *S. aureus ΔbioY*. (A, B) Growth of *S. aureus* JE2 and *ΔbioY* in TSB and biotin saturated sTMS. *S. aureus* JE2 and *ΔbioY* was inoculated to an OD_{600} = 0.0001 in 500 µl Tryptic Soy Broth (TSB) (A) or in sTMS with 100 nM biotin (B) in a 48-well plate. Growth was monitored every 15 min for 20 h at 37°C using an Epoch2 orbital reader. For reasons of clarity, only values taken every 1 h (A) and 2 h (B) are displayed. Mean and SD of three independent experiments are shown. (C, D) Growth of *S. aureus ΔbioY* in SNM20 and SNM20 with 4 nM biotin. *S. aureus* JE2 and *ΔbioY* was inoculated to an OD_{600} = 0.0001 in 500 µl biotin-deficient 20x Synthetic Nasal Medium (SNM20) with (D) and without (C) 4 nM biotin (see Suppl. Methods) in a 48-well plate. Growth was monitored every 15 min for 40 h at 37°C using an Epoch2 orbital reader. For reasons of clarity, only values taken every 2.5 h are displayed. Mean and SD of three independent experiments are shown. (E) Autolysis assay. Strains grown to mid-log phase were washed in PBS and treated with 0.025% Triton X100. The JE2 *tagO* mutant (Δ*tagO*) was used as a positive control. Lysis was monitored every 15 min for 4 h using an Epoch2 orbital reader. Mean and SD of three independent experiments are shown. (F) Biotin levels in culture supernatants in SNM20. Biotin levels of strains grown for 24 h in 10 ml biotin-deficient 20x Synthetic Nasal Medium (SNM20) were assessed using the IDK Biotin ELISA Kit (K8141) from Immundiagnostik. Mean and SD of three independent experiments are shown. Filled circles indicate measurable biotin levels in the supernatants. Filled squares indicate samples in which biotin levels were too low for quantification. (G) Growth support of *ΔbioA* by *ΔbioY* supernatants. *ΔbioA* was inoculated to an OD_{600} = 0.0001 in 500 µl biotin-deficient 20x Synthetic Nasal Medium (SNM20) containing 50% sterile filtered *S. aureus* USA300 JE2 WT and *ΔbioY* supernatants (grown in SNM20) or 4 nM biotin and growth was monitored in 48-well plates for 40 h at 37°C using an Epoch2 orbital reader. For reasons of clarity, only values taken every 2.5 h are displayed. Mean and SD of three independent experiments are shown.

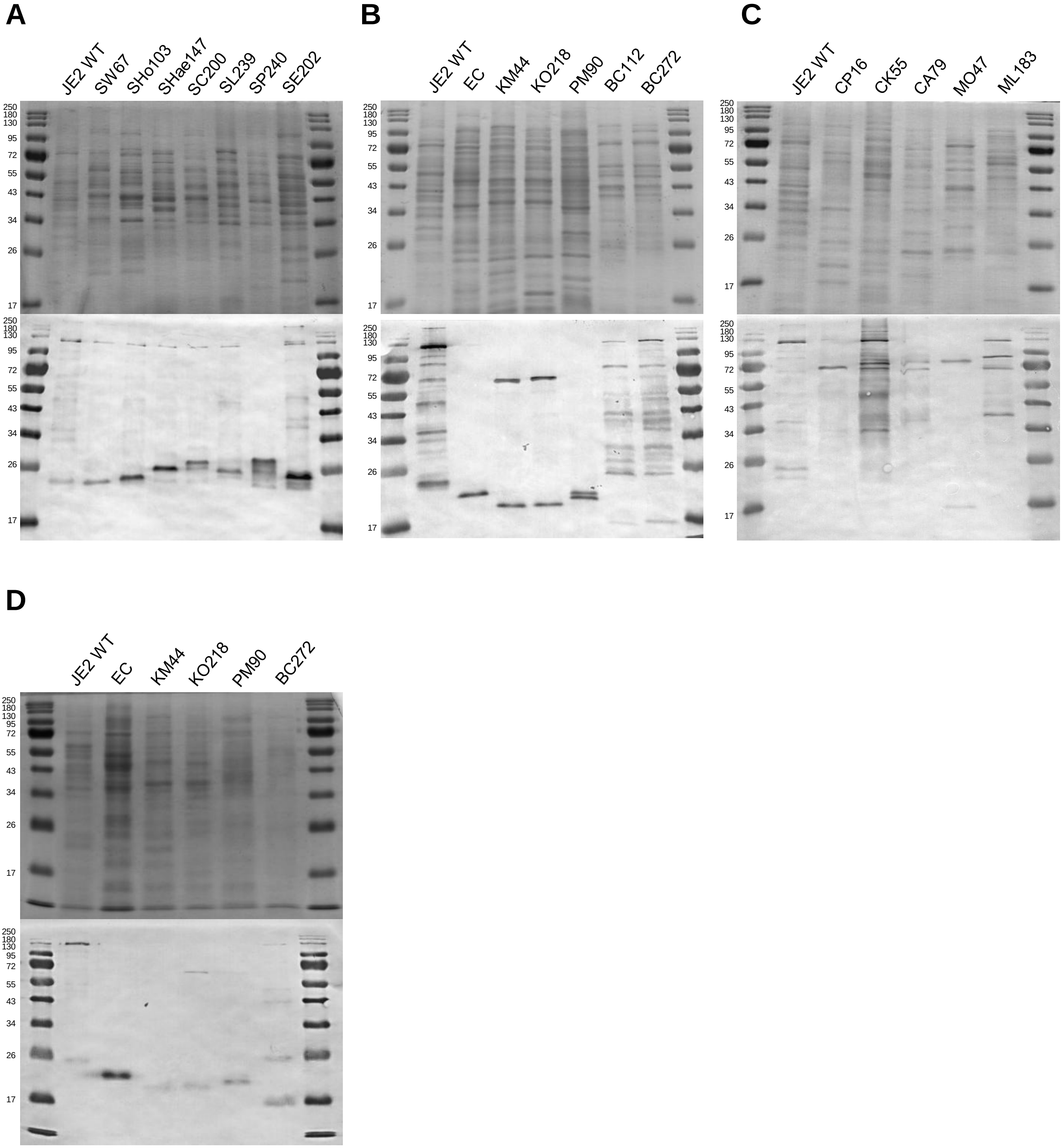

Supplementary Figure 3: Biotinylated enzymes in nasal isolates grown in full media or in sTMS. Streptavidin blots (bottom) and SDS-Page (top) of crude extracts of different species grown in BHI-T overnight (A-C) or 24h in sTMS (D). Biotinylated enzymes were detected using the alkaline phosphatase activity of the streptavidin-AP conjugate. JE2 WT: *S. aureus* USA300 JE2 WT; SW67: *S. warneri* 67; SHo103: *S. hominis* 103; SHae147: *S. haemolyticus* 147; SC200: *S. capitis* 200; SL239: *S. lugdunensis* 239; SP240: *S. pettenkoferi* 240; SE202: *S. epidermidis* 202; EC: *E. coli* BW25113 WT, KM44*: K. michiganensis* 44*;* KO218*: K. oxytoca* 218*;* PM90: *P. mirabilis* 90*;* BC112*: B. cereus* 112*;* BC272*: B. cereus* 272*;* CP16*: C. propinquum* 16*;* CK55*: C. kefirresidentii* 55*;* CA79*: C. accolens* 79*;* MO47*: M. osloensis* 47, and ML183*: M. luteus* 183*.* Color Prestained Protein Standard from NEB (P7719S) was used as standard.

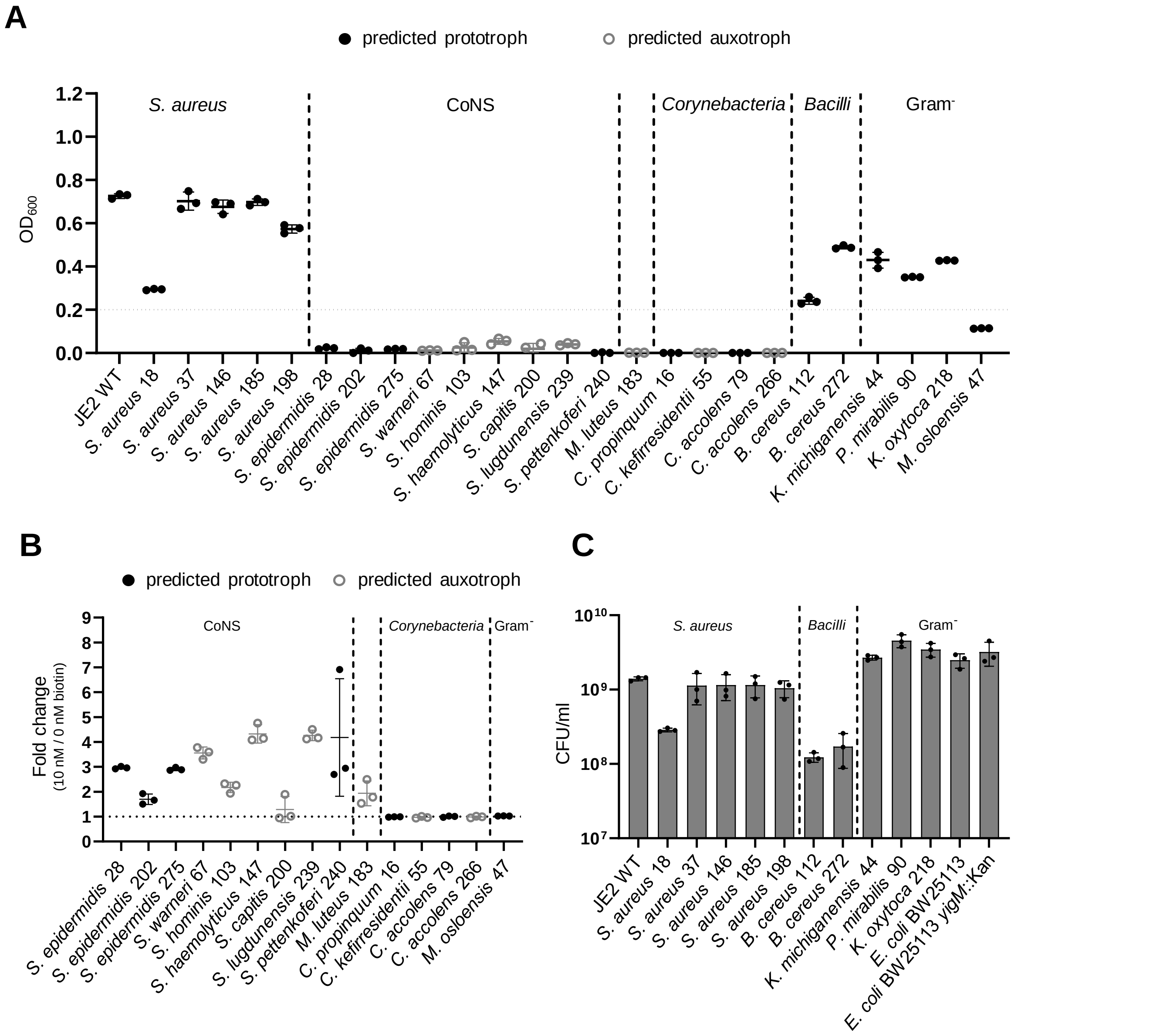

Supplementary Figure 4: Biotin-dependent growth of nasal isolates in sTMS with and without biotin. (A) Growth of nasal isolates in sTMS. Strains were inoculated to an OD_{600} = 0.0001 in 500 µl sTMS and OD_{600} was measured after 30 h growth at 37°C using an Epoch2 orbital reader. Threshold at OD_{600} = 0.2 was used as indication for growth. Mean and SD of three independent experiments are shown. (B) Impact of biotin supplementation. Bacterial species which did not show grow in Suppl. Fig. 4A were inoculated to an OD_{600} = 0.0001 in 500 µl sTMS with either 0 nM or 10 nM biotin and fold change at OD_{600} was determined after 30 h growth at 37°C in an Epoch2 orbital reader. Mean and SD of three experiments are shown. (C) CFUs of prototrophic nasal isolates grown for 24 h in sTMS. CFUs were determined by plating serial dilutions of cultures grown for 24 h in sTMS. Mean and SD of three independent experiments are shown.

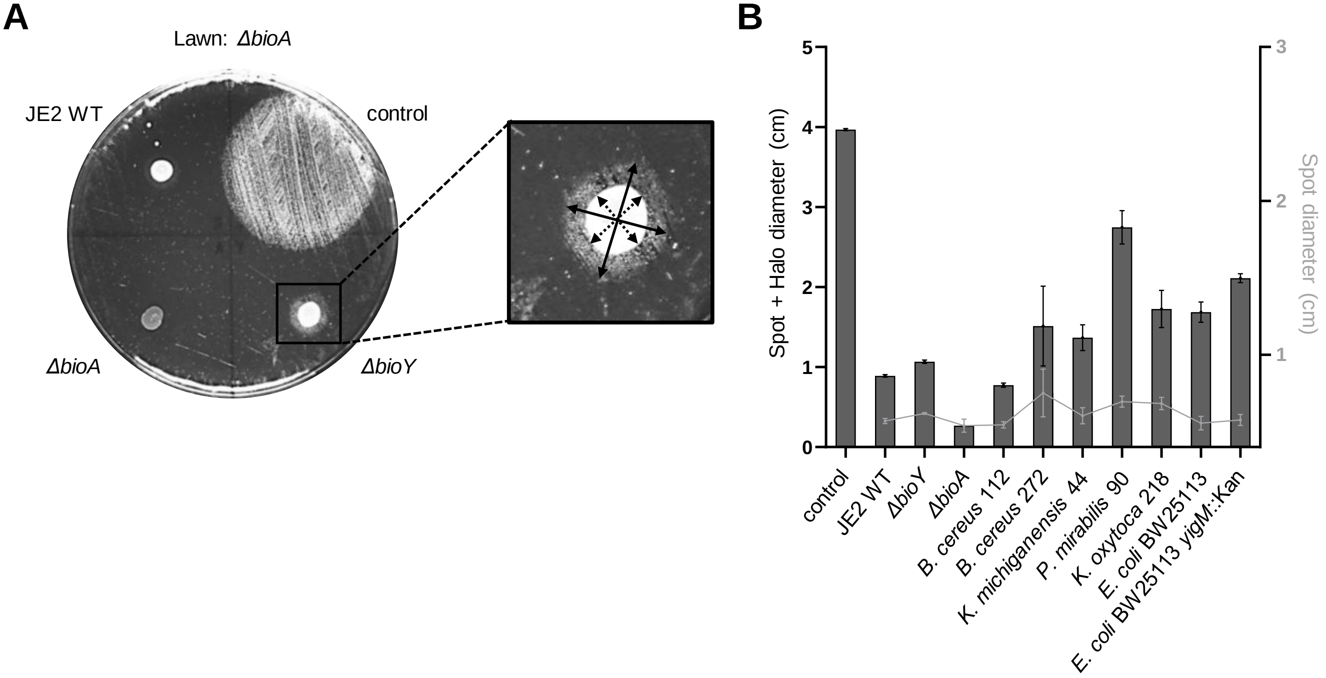

Supplementary Figure 5: Growth of *ΔbioA* on sTMS agar inoculated with nasal commensals. (A, B) Bacterial suspension of the strain *ΔbioA* was adjusted to an OD_{600} = 0.05 and applied to sTMS plates to form a lawn using cotton swabs. Nasal commensal strains were adjusted to an OD_{600} = 5 and 5 µl were spotted on the plates. For control 1 µl of 100 µM biotin was spotted on the lawn. Plates were incubated at 37°C for 48 h and the diameter of growth of *ΔbioA* surrounding the spots was measured as indicated using ImageJ. (A) Representative plate. The spot diameters (dotted arrows) and the spot with halo diameters (solid arrows) were measured and the mean of each was calculated. (B) Summary of growth of *ΔbioA* in the presence of various nasal commensals. Shown are the mean and SD of three independent experiments described in A (exception: control was only performed twice).

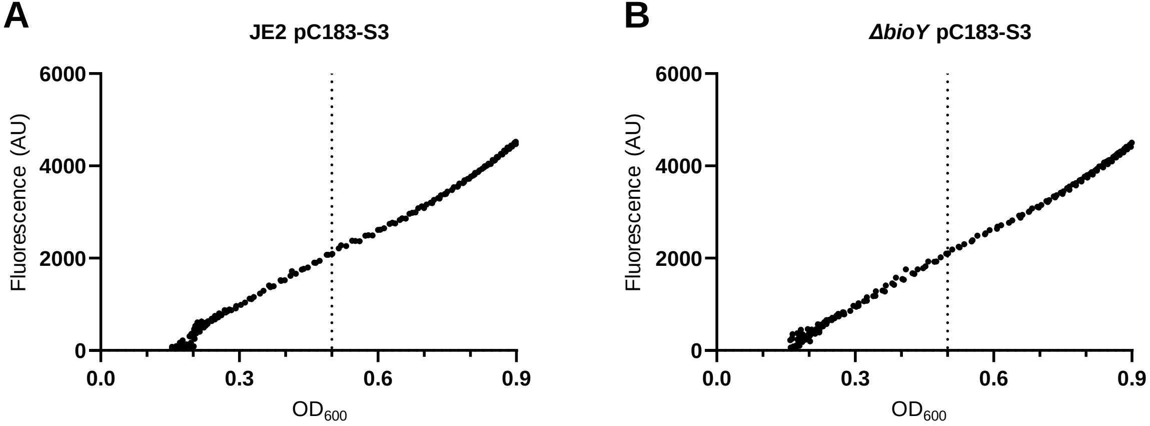

Supplementary Figure 6: GFP/OD correlation of fluorescent *S. aureus* strains. Fluorescently labeled *S. aureus* strains (A) JE2 pC183-S3 and (B) Δ*bioY* pC183-S3 were inoculated in biological triplicates to an OD_{600}= 0.0001 in sTMS in 48-well microplates. Growth was monitored by measuring the OD_{600} and fluorescence (Ex. 480 nm and Em. 520 nm) every 15 min for 48 h at 30°C in a BioTek Synergy H1 Reader (Agilent).

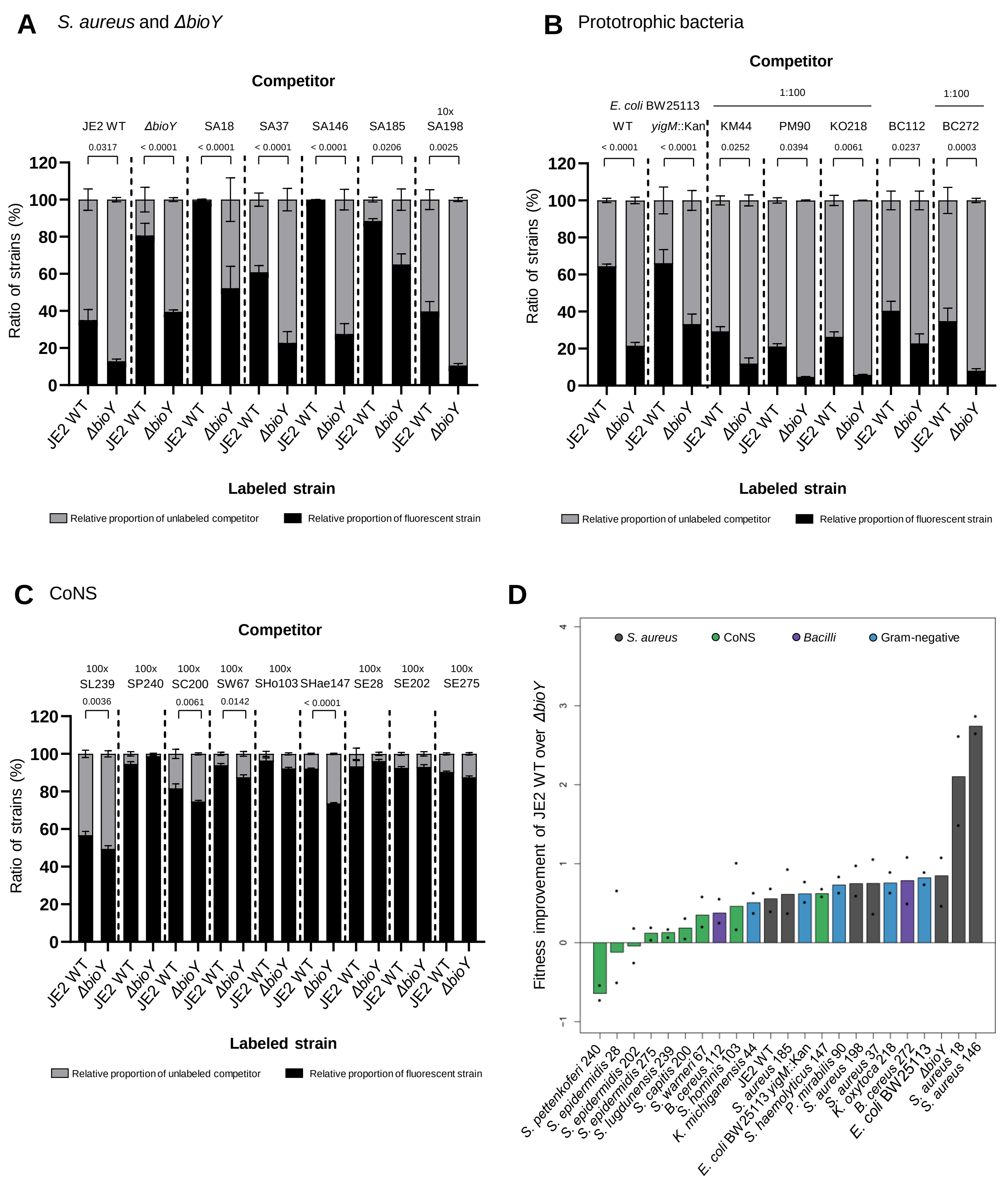

Supplementary Figure 7: Co-cultivation of JE2 WT and *ΔbioY* with different commensals in sTMS. Fluorescently labeled JE2 WT (JE2 pC183-S3) and *ΔbioY* (*ΔbioY* pC183-S3) were inoculated to an OD_{600} = 0.0001 in 500 µl sTMS cultivated in coculture with (A) *S. aureus* (JE2 WT, JE2 *ΔbioY,* SA18: *S. aureus* 18, SA37: *S. aureus* 37, SA146: *S. aureus* 146, SA185: *S. aureus* 185, and SA198: *S. aureus* 198), (B) with biotin prototrophic nasal isolates (*E. coli* BW25113 WT, *E. coli* BW25113 *yigM*::Kan, KM44*: K. michiganensis* 44*;* PM90*: P. mirabilis* 90*;* KO218*: K. oxytoca* 218*;* BC112*: B. cereus* 112*;* BC272*: B. cereus* 272) or (C) with nasal CoNS (SL239: *S. lugdunensis* 239; SP240: *S. pettenkoferi* 240; SC200: *S. capitis* 200; SW67: *S. warneri* 67; SHo103: *S. hominis* 103; SHae147: *S. haemolyticus* 147; SE28: *S. epidermidis* 28; SE202: *S. epidermidis* 202; SE275: *S. epidermidis* 275) for 40 h at 30°C. Depending on the competitor different ratio (1:1, 10x, 100x and 1:100) were tested. Growth was monitored by measuring the OD_{600} and fluorescence intensity of the GFP signal (Em. 480 nm, Ex. 520 nm) using a BioTek Synergy H1 Reader (Agilent). Relative proportion of labeled *S. aureus* strains and unlabeled competitors was determined by calculating the ratio of the fluorescence and OD_{600}. For all figures mean and SD of three independent experiments are shown. Statistical analysis was performed using two-way ANOVA (P < 0.0001) with subsequent multiple comparison by comparing all relative proportions of *S. aureus* Je2 pC183-S3 and *S. aureus* Je2 *ΔbioY* pC183-S3 of each mixed culture with the shared labor condition (D) Sorted fitness improvement of JE2 WT over JE2 Δ*bioY* against competitor strains is displayed as residual (log10) of the determined fitness to the assumed line of equal fitness in Figure 6A. Positive values indicate improved fitness of JE2 WT, negative values reduced fitness. Bars represent the mean metric of fitness improvement. Dots represent maximal or minimal values, obtained if residuals are calculated for each combination of biological replicates individually.

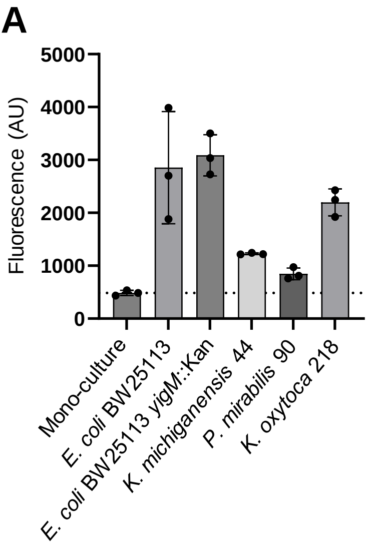

Supplementary Figure 8: Co-cultivation of Δ*bioA* with prototrophic commensals. Fluorescently labeled *S. aureus* JE2 *ΔbioA* (*ΔbioA* pC183-S3) was inoculated to an OD_{600} = 0.0001 in 500 µl sTMS cultivated in coculture with prototrophic strains *E. coli* BW25113 WT (1:1), *E. coli* BW25113 *yigM*::Kan (1:1), *K. michiganensis* 44 (1:100), *P. mirabilis* 90 (1:100), and *K. oxytoca* 218 (1:100) for 40 h at 30°C. Growth was monitored by measuring the fluorescence intensity of the GFP signal (Em. 480 nm, Ex. 520 nm) of *ΔbioA* pC183-S3 at timepoint 40 h using a BioTek Synergy H1 Reader (Agilent).

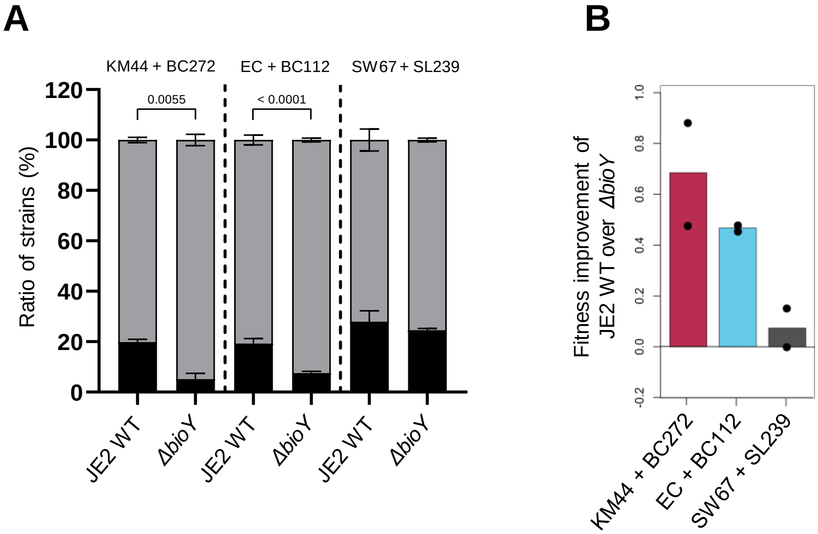

Supplementary Figure 9: Triple co-cultivation of JE2 WT and *ΔbioY* with different commensals in sTMS. Fluorescently labeled JE2 WT (JE2 pC183-S3) and *ΔbioY* (*ΔbioY* pC183-S3) were inoculated to an OD_{600} of 0.0001 in 500 µl sTMS cultivated in coculture with *E. coli* BW25113 WT (EC) and *B. cereus* 112 (BC112) (OD 0.0001 for each commensal), *K. michiganensis* 44 (KM44) and *B. cereus* 272 (BC272) (OD 0.000001 for each commensal), and *S. warneri* 67 (SW67) together with *S. lugdunensis* 239 (SL239) (OD 0.01) for 40 h at 30°C. Growth was monitored by measuring the OD_{600} and fluorescence intensity of the GFP signal (Em. 480 nm, Ex. 520 nm) using a BioTek Synergy H1 Reader (Agilent). Relative proportion of labeled *S. aureus* strains and unlabeled competitors was determined by calculating the ratio of the fluorescence and OD_{600}. For all figures mean and SD of three independent experiments are shown. Statistical analysis was performed using two-way ANOVA ($P < 0.0001$) with subsequent multiple comparison by comparing all relative proportions of *S. aureus* Je2 pC183-S3 and *S. aureus* Je2 *ΔbioY* pC183-S3 of each mixed culture with the shared labor condition. (B) Fitness improvement as determined in (Suppl. Figure 7D) of JE2 WT over JE2 Δ*bioY* against a mixture of two competitor strains. Positive values indicate improved fitness of JE2 WT, negative values reduced fitness. Bars represent the mean metric of fitness improvement. Dots represent maximal or minimal values, obtained if residuals are calculated for each combination of biological replicates individually.
