# Supplementary material for: Competitive fitness of Staphylococcus aureus against nasal commensals depends on biotin biosynthesis and acquisition
Source: ISME J. 2025 Nov 4;19(1):wraf248. doi: 10.1093/ismejo/wraf248 (PMC12642757; doi:10.1093/ismejo/wraf248)
Supplement: 3_Supplementary_Tables_wraf248 [file 3_supplementary_tables_wraf248.docx]

**Table S1: Bacterial strains.**

| **Strain** | **Additional Information** | **Reference** |
| --- | --- | --- |
| *Escherichia coli* IMO8B | Plasmid artificial modification (PAM) strain for *S. aureus* CC8 strains | [1] |
| *Staphylococcus aureus* USA300 JE2 | Wildtype | [2] |
| *Staphylococcus aureus* USA300 JE2 *pyc::Erm* | Transposon mutant of the pyruvate carboxylase (NE754) from the Nebraska Transposon Mutant Library (NTML) | [2] |
| *Staphylococcus aureus* USA300 JE2 *ΔbioA* | Markerless deletion of *bioA* gene; biotin biosynthesis mutant | This study |
| *Staphylococcus aureus* USA300 JE2 *ΔbioA::bioA* | *bioA* genomic complementation with introduced silent mutation for *BamHI* | This study |
| *Staphylococcus aureus* USA300 JE2 *ΔbioY* | Markerless deletion of *bioY* gene; biotin uptake mutant | This study |
| *Staphylococcus aureus* USA300 JE2 *ΔbioY::bioY* | *bioY* genomic complementation with introduced silent mutation for *SmaI* | This study |
| *Staphylococcus aureus* USA300 JE2 *ΔtagO* | Markerless deletion of *tagO* gene; unable to synthesis the first step of the WTA biosynthesis | [3] |
| *Staphylococcus aureus* USA300 JE2 pC183-S3 | Wildtype strain containing GFP-expressing plasmid | This study |
| *Staphylococcus aureus* USA300 JE2 *ΔbioA* pC183-S3 | *ΔbioA* strain containing GFP-expressing plasmid  Resistance and maintenance: 12.5 µg/ml tetracycline | This study |
| *Staphylococcus aureus* USA300 JE2 *ΔbioY* pT183-S3 | *ΔbioY* strain containing GFP-expressing plasmid  Resistance and maintenance: 12.5 µg/ml tetracycline | This study |
| *Escherichia coli* BW25113 | Wildtype | [4] |
| *Escherichia coli* BW25113 *yigM::*Kan | Biotin transporter mutant from the Keio collection (JW3803) | [4] |
| *Staphylococcus epidermidis* SE28 (Strain 28) | Nasal isolate | [5] |
| *Staphylococcus epidermidis* SE202 (Strain 202) | Nasal isolate | [5] |
| *Staphylococcus epidermidis* SE275 (Strain 275) | Nasal isolate | [5] |
| *Staphylococcus pettenkoferi* SP240 (Strain 240) | Nasal isolate | [5] |
| *Staphylococcus hominis* SHo103 (Strain 103) | Nasal isolate | [5] |
| *Staphylococcus haemolyticus* SHae147 (Strain 147) | Nasal isolate | [5] |
| *Staphylococcus warneri* SW67 (Strain 67) | Nasal isolate | [5] |
| *Staphylococcus capitis* SC200 (Strain 200) | Nasal isolate | [5] |
| *Staphylococcus lugdunensis* SL239 (Strain 239) | Nasal isolate | [5] |
| *Bacillus cereus* BC112 (Strain 112) | Nasal isolate | [5] |
| *Bacillus cereus* BC272 (Strain 272) | Nasal isolate | [5] |
| *Klebsiella oxytoca* KO218 (Strain 218) | Nasal isolate | [5] |
| *Klebsiella michiganensis* KM44 (Strain 44) | Nasal isolate | [5] |
| *Proteus mirabilis* PM90 (Strain 90) | Nasal isolate | [5] |
| *Moraxella osloensis* MO47 (Strain 47) | Nasal isolate | [5] |
| *Corynebacterium propinquum* CP16 (Strain 16) | Nasal isolate | [5] |
| *Corynebacterium kefirresidentii* CK55 (Strain 55) | Nasal isolate | [5] |
| *Corynebacterium accolens* CA79 (Strain 79) | Nasal isolate | [5] |
| *Corynebacterium accolens* CA266 (Strain 266) | Nasal isolate | [5] |

**Table S2: Oligonucleotides and Plasmids.**

| **Oligonucleotides** | | | |
| --- | --- | --- | --- |
| **Name** | **5‘-3‘ Sequence** | **Purpose** | **References** |
| SeqFW_bioA | CACGTGGTCATCGAGTTTG | Primer (forward) for screening of *ΔbioA* in *S. aureus* USA300 JE2 | This study |
| SeqRV_bioA | TATAACTATGCGTTGTCACG | Primer (reverse) for screening of *ΔbioA* in *S. aureus* USA300 JE2 | This study |
| A:KO_bioA_KpnI | AACGCCGGTACCGGACGTTTCC | Primer A for creating *ΔbioA* fragment to clone in pIMAY  Restriction site: *KpnI* | This study |
| B:KO_bioA_ATG | CATTTTTAACTCCTATTAATTG | Primer B for creating *ΔbioA* fragment to clone in pIMAY | This study |
| C:KO_bioA_TAA | GAGTTAAAAATGTAATATGAATTTGGCTAAAC | Primer C for creating *ΔbioA* fragment to clone in pIMAY | This study |
| D:KO_bioA_SacI | TAATATTGTGGTGAGCTCTGTCTACAC | Primer D for creating *ΔbioA* fragment to clone in pIMAY  Restriction site: *SacI* | This study |
| B_R:compl_JE2dbioA_BamHI | GAATATTTGAGGATCCTAGCAG | Primer B_R for creating the complementation fragment of *bioA* and to clone in pIMAY  Restriction site: *BamHI* (in *bioA*) | This study |
| C_F:compl_JE2dbioA_BamHI | CTGCTAGGATCCTCAAATATTC | Primer C_F for creating the complementation fragment of *bioA* and to clone in pIMAY  Restriction site: *BamHI* (in *bioA*) | This study |
| SeqFW_BioY | TTTACCAAAAGGAAATGTTGAAG | Primer (forward) for screening of *ΔbioY* in *S. aureus* USA300 JE2 | This study |
| SeqRV_BioY | AAGCGTTTAATCACATCTTTTTG | Primer (reverse) for screening of *ΔbioY* in *S. aureus* USA300 JE2 | This study |
| A: KO_BioY_KpnI | ATATTGGGTACCAGAAGCGGTTAAAATTG | Primer A for creating *ΔbioY* fragment to clone in pIMAY  Restriction site: *KpnI* | This study |
| B: KO_BioY_ATG | CATTTTTTACTTCCTTTCGTATTTC | Primer B for creating *ΔbioY* fragment to clone in pIMAY | This study |
| C: KO_BioY_TAA | GTAAAAAATGTAATCATATTTAAGATAGTAAAG | Primer C for creating *ΔbioY* fragment to clone in pIMAY | This study |
| D: KO_BioY_SacI | ATGTGGAGCTCCATAGTCTTTTCC | Primer D for creating *ΔbioY* and complementation fragment to clone in pIMAY  Restriction site: *SacI* | This study |
| A2:compl_bioY_EcoRV | ATTGGGATATCGAAGCGGTTAAAATTG | Primer A2 for creating the complementation fragment of *bioY* and to clone in pIMAY  Restriction site: *EcoRV* | This study |
| B_R: compl_JE2dbioY_SmaI | CTTTTAATATATCCCCGGGCAAATAAG | Primer B_R for creating the complementation fragment of *bioY* and to clone in pIMAY  Restriction site: *SmaI* (in *bioY*) | This study |
| C_F: compl_JE2dbioY_SmaI | CTTATTTGCCCGGGGATATATTAAAAG | Primer C_F for creating the complementation fragment of *bioY* and to clone in pIMAY  Restriction site: *SmaI* (in *bioY*) | This study |
| qPCR_F:bioA | AGCAGTTGGTTTTGGGAGAA | Forward *bioA* primer for qPCR | This study |
| qPCR_R:bioA | TTCACACCATGCGAATCACT | Reverse *bioA* primer for qPCR | This study |
| qPCR_F:gyrB_SA | GGTGCTGGGCAAATACAAGT | Forward *gyrB* primer for qPCR (housekeeping gene) | This study |
| qPCR_R:gyrB_SA | TGGGATACCACGTCCGTTAT | Reverse *gyrB* primer for qPCR (housekeeping gene) | This study |
| **Plasmids** | | | |
| pIMAY (empty vector) | N.A. | *E. coli* / Staphylococcus shuttle vector. Thermosensitive vector for allelic exchange 10 µg/ml chloramphenicol | [6] |
| pIMAY:*ΔbioA* | N.A. | Plasmid for the markerless deletion of *ΔbioA* in *S. aureus* | This study |
| pIMAY:compl_*bioA* | N.A. | Plasmid for the genomic complementation of *ΔbioA* in *S. aureus* | This study |
| pIMAY:*ΔbioY* | N.A. | Plasmid for the markerless deletion of *ΔbioY* in *S. aureus* | This study |
| pIMAY:compl_*bioY* | N.A. | Plasmid for the genomic complementation of *ΔbioY* in *S. aureus* | This study |
| pC183-S3 | N.A. | GFP-expressing *S. aureus* plasmid, pC183 is a derivate of pCX19  antibiotic: 12.5 µg/ml tetracycline | [7] |

* Restriction sites are underlined

**Table S3: In silico screening for biotin biosynthesis and uptake genes.**

| **AA sequence of gene (Query Covery - Per. Identity)** | | | | | | |
| --- | --- | --- | --- | --- | --- | --- |
|  | **Biosynthesis** | | | | **Transporter** | |
|  | **BioD** | **BioA/BioK** | **BioB** | **BioF** | **BioY** | **YigM/BioP** |
| ***S. aureus* USA300 FPR3757** | SAUSA300_2373 (100% - 100%) | SAUSA300_2372 (100% - 100%) | SAUSA300_2371 (100% - 100%) | SAUSA300_2370 (100% - 100%) | SAUSA300_2233 (100% - 100%) | not present |
| ***S. aureus* 18** | KNBGPNCC_02081 (100% - 97.37%) | KNBGPNCC_02082 (100% - 99.34%) | KNBGPNCC_02083 (100% - 99.40%) | KNBGPNCC_02084 (100% - 97.04%) | KNBGPNCC_02224 (100% - 99.46%) | not present |
| ***S. aureus* 37** | EGPHAPLG_02120 (100% - 98.25%) | EGPHAPLG_02121 (100% - 99.12%) | EGPHAPLG_02122 (100% - 99.70%) | EGPHAPLG_02123 (100% - 99.73%) | EGPHAPLG_02264 (100% - 98.91%) | not present |
| ***S. aureus* 146** | CBKCCEEI_02200 (100% - 98.68%) | CBKCCEEI_02201 (100% - 99.34%) | CBKCCEEI_02202 (100% - 99.70%) | CBKCCEEI_02203 (100% - 99.73%) | CBKCCEEI_02344 (100% - 98.91%) | not present |
| ***S. aureus* 185** | MLNDHHGH_02120 (100% - 99.12%) | MLNDHHGH_02121 (100% - 99.78%) | MLNDHHGH_02122 (100% - 99.40%) | MLNDHHGH_02123 (100% - 99.19%) | MLNDHHGH_02261 (100% - 99.46%) | not present |
| ***S. aureus* 198** | NGNHBJHK_01671 (100% - 99.12%) | NGNHBJHK_01672 (100% - 99.56%) | NGNHBJHK_01673 (100% - 99.70%) | NGNHBJHK_01674 (100% - 98.65%) | NGNHBJHK_01814 (100% - 100%) | not present |
| ***S. epidermidis* 28** | OANAEING_00106 (97% - 65.32%) | OANAEING_00107 (100% - 77.16%) | OANAEING_00152 (95% - 77.67%) | OANAEING_00108 (100% - 53.37%) | OANAEING_00591 (98% - 69.61%) | not present |
| ***S. epidermidis* 202** | ICJJFBAN_00085 (97% - 65.32%) | ICJJFBAN_00086 (100% - 76.72%) | ICJJFBAN_00133 (95% - 77.99%) | ICJJFBAN_00087 (100% - 53.10%) | ICJJFBAN_00566 (98% - 69.91%) | not present |
| ***S. epidermidis* 275** | PILPMCFO_00102 (97% - 65.32%) | PILPMCFO_00103 (100% - 76.94%) | PILPMCFO_00151 (95% - 77.99%) | PILPMCFO_00104 (100% - 53.10%) | PILPMCFO_00591 (98% - 69.91%) | not present |
| ***S. pettenkoferi* 240** | FKIELEFI_00251 (98% - 55.36%) | FKIELEFI_00252 (97% - 75.68%) | FKIELEFI_00601 (96% - 80.50%) | FKIELEFI_00253 (95% - 49.01%) | FKIELEFI_00674 (98% - 67.96%) | not present |
| ***S. hominis* 103** | not present | not present | LOKDFPLI_00182 (95% - 76.10%) | not present | LOKDFPLI_00571 (98% - 73.48%) | not present |
| ***S. haemolyticus* 147** | not present | not present | MPOGNJFB_00216 (95% - 76.42%) | not present | MPOGNJFB_00689 (98% - 70.72%) | not present |
| ***S. warneri* 67** | not present | not present | HDMMFILG_01782 (95% - 77.99%) | not present | HDMMFILG_00597 (98% - 66.85%) | not present |
| ***S. capitis* 200** | not present | not present | MPEAPJOH_00130 (95% - 77.67%) | not present | MPEAPJOH_00633 (98% - 68.51%) | not present |
| ***S. lugdunensis* 239** | not present | not present | OLENELJG_01701 (95% - 75.24%) | not present | OLENELJG_02216 (98% - 65.75%) | not present |
| ***B. cereus* 112** | BKHCPAHN_04114 (85% - 25.63%) | BKHCPAHN_04115 (99% - 50.44%) | BKHCPAHN_04110 (94% - 52.37%) | BKHCPAHN_04113 (97% - 31.54%) | BKHCPAHN_03519 (98% - 41.44%); BKHCPAHN_04943 (82% - 27.63%) | not present |
| ***B. cereus* 272** | AKMJOJEE_05182 (52% - 31.40%) | AKMJOJEE_05181 (99% - 50.00%) | AKMJOJEE_05186 (94% - 52.37%) | AKMJOJEE_05183 (99% - 31.95%) | AKMJOJEE_00330 (98% - 41.99%) | not present |
| ***K. oxytoca* 218** | HDCOIJDD_02456 (50% - 27.67%); HDCOIJDD_03662 (71% - 29.21%) | HDCOIJDD_03666 (96% - 31.96%) | HDCOIJDD_03665 (90% - 33.01%) | HDCOIJDD_03664 (92% - 27.04%) | not present | HDCOIJDD_05288 (96% - 53.99%) |
| ***K. michiganensis* 44** | BIOMNINF_02536 (50% - 28.93%); BIOMNINF_03926 (71% - 28.65%) | BIOMNINF_03930 (96% - 31.51%) | BIOMNINF_03929 (90% - 33.33%) | BIOMNINF_03616 (57% - 25.88%); BIOMNINF_03928 (99% - 25.52%) | not present | BIOMNINF_05442 (96% - 53.26%) |
| ***P. mirabilis* 90** | PIANBNLJ_01114 (78% - 26.84%); PIANBNLJ_01689 (50% - 27.59%) | PIANBNLJ_01110 (96% - 35.24%) | PIANBNLJ_01111 (85% - 33.33%) | PIANBNLJ_01112 (95% - 24.45%) | not present | PIANBNLJ_00391 (97% - 54.48%) |
| ***M. osloensis* 47** | MIFPPGHA_00391 (93% - 23.56%) | MIFPPGHA_00759 (96% - 33.56%) | MIFPPGHA_01889 (84% - 33.68%) | MIFPPGHA_01890 (99% - 27.18%) | not present | not present |
| ***C. propinquum* 16** | GOMOEIOP_00670 (53% - 27.20%) | GOMOEIOP_00671 (97% - 28.69%) | GOMOEIOP_01077 (93% - 32.59%) | GOMOEIOP_00151 (90% - 29.64%) | not present | not present |
| ***C. kefirresidentii* 55** | KMHAEAEI_01944 (67% - 30.92%) | KMHAEAEI_01945 (98% - 29.49%) | KMHAEAEI_00101 (93% - 33.12%) | not present | KMHAEAEI_01439 (96% - 33.33%) | not present |
| ***C. accolens* 79** | KBHGJMGA_01921 (68% - 31.82%) | KBHGJMGA_01922 (98% - 29.05%) | KBHGJMGA_00078 (93% - 33.12%) | KBHGJMGA_01729 (98% - 28.79%) | KBHGJMGA_01467 (54% - 39.00%) | not present |
| ***C. accolens* 266** | GPDOMJLF_00455 (57% - 31.78%) | GPDOMJLF_00454 (98% - 28.76%) | GPDOMJLF_00061 (93% - 33.44%) | not present | GPDOMJLF_00904 (97% - 31.11%) | not present |
| ***D. pigrum* 61** | not present | not present | not present | not present | DBINHAEK_00162 (98% - 31.22%) | not present |
| ***M. luteus* 183** | not present | not present | FMEKCACA_01986 (94% - 43.22%) | not present | FMEKCACA_01526 (92% - 41.24%) | not present |
| ***F. magna* 56** | not present | not present | not present | not present | OMHFFEHG_00592 (90% - 29.51%) | not present |
| ***F. magna* 260** | not present | not present | FMMKNMHA_00570 (91% -21.82%) | not present | FMMKNMHA_00704 (94% - 30.00%) | not present |
| ***C. acnes* 226** | PFKGMPBN_00975 (50% - 30.00%) | PFKGMPBN_00974 (89% - 29.73%) | PFKGMPBN_01429 (94% - 40.51%) | PFKGMPBN_01865 (89% - 27.38%) | PFKGMPBN_01419 (98% - 28.27%) | not present |
| ***C. avidum* 88** | OHKOEAGF_01439 (50% - 30.83%) | OHKOEAGF_01440 (89% - 27.72%) | OHKOEAGF_01017 (94% - 41.46%) | OHKOEAGF_01914 (87% - 25.30%) | OHKOEAGF_01029 (98% - 29.84%) | not present |
| ***C. granulosum* 214** | MPFDHIFL_01315 (67% - 28.57%) | MPFDHIFL_01316 (96% - 30.34%) | MPFDHIFL_01138 (95% - 39.66%) | MPFDHIFL_01723 (96% - 27.96%) | MPFDHIFL_01129 (57% - 28.57%) |  |

**References:**

1. Monk IR, Tree JJ, Howden BP, *et al.* Complete bypass of restriction systems for major *Staphylococcus aureus* lineages. *mBio* 2015;**6**:e00308-15. DOI: 10.1128/mBio.00308-15

2. Fey PD, Endres JL, Yajjala VK, *et al.* A genetic resource for rapid and comprehensive phenotype screening of nonessential *Staphylococcus aureus* genes. *mBio* 2013;**4**:e00537-12. DOI: 10.1128/mBio.00537-12

3. Krusche J, Beck C, Lehmann E, *et al.* Characterization and host range prediction of *Staphylococcus aureus* phages through receptor-binding protein analysis. *Cell Reports* 2025;**44**:115369. DOI: 10.1016/j.celrep.2025.115369

4. Baba T, Ara T, Hasegawa M, *et al.* Construction of *Escherichia coli* K‐12 in‐frame, single‐gene knockout mutants: the Keio collection. *Mol Sys Biol* 2006;**2**:2006.0008. DOI: 10.1038/msb4100050

5. Camus L, Franz J, Gerlach D, *et al.* Tyrosine availability shapes *Staphylococcus aureus* nasal colonization and interactions with commensal communities. 2025. Microbiology, 2025.

6. Monk IR, Shah IM, Xu M, *et al.* Transforming the untransformable: Application of direct transformation to manipulate genetically *Staphylococcus aureus* and *Staphylococcus epidermidis*. *mBio* 2012;**3**:e00277-11. DOI: 10.1128/mBio.00277-11

7. Hussain M, Becker K, Von Eiff C, *et al.* Identification and characterization of a novel 38.5-kilodalton cell surface protein of *Staphylococcus aureus* with extended-spectrum binding activity for extracellular matrix and plasma proteins. *J Bacteriol* 2001;**183**:6778–6786. DOI: 10.1128/JB.183.23.6778-6786.2001
