# Supplementary material for: Competitive fitness of Staphylococcus aureus against nasal commensals depends on biotin biosynthesis and acquisition
Source: ISME J. 2025 Nov 4;19(1):wraf248. doi: 10.1093/ismejo/wraf248 (PMC12642757; doi:10.1093/ismejo/wraf248)
Supplement: Supplementary_Methods [file supplementary_methods.docx]

**Biotin-deficient Synthetic Nasal Medium (SNM20)**

Synthetic Nasal Medium (SNM) was prepared in 20x concentration (SNM20) as described previously with the exception of lacking biotin [1, 2].

**Growth curves in TSB and SNM20**

Generation of the inoculum: *S. aureus* USA300 JE2 and mutants were grown to stationary phase overnight at 37°C with agitation. Cultures were centrifuged for 1 min at 15871 rcf and washed once with PBS. Cells were adjusted to OD_600_= 0.1 and 1 µl was used to inoculate 1 ml of TSB or SNM20 (final OD_600_= 0.0001).

Supplementation with biotin: When appropriate, final biotin concentrations of 4 nM were added using 1 µl or 1000-fold concentrated stock solutions to 1 ml of sTMS. For generation of spent medium in SNM20, *S. aureus* USA300 JE2 and Δ*bioY* mutant were grown to stationary phase overnight in BHI-T at 37°C with agitation, harvested by centrifugation (15871 rcf) and washed once with PBS. 10 ml SNM20 medium were inoculated to an OD_600_ of 0.05 and grown for 24 h (stationary phase) at 37°C with agitation. Cells were removed by centrifugation (10 min at 3197 rcf), the supernatants were collected and sterile filtered using a 0.45 µM Millex‑HA filter (SLHA033SB). Sterility was confirmed by plating of 200 µl on TSA plates supernatants were stored until use at 4°C. Spent medium was diluted to 50% by mixing 250 µl with 750 µl fresh SNM20.

Growth curve generation: After inoculation 500 µl of bacterial suspension was transferred to a 48-well microplate (Nunc, Thermo Scientific) and growth was monitored automatically by measuring the OD_600_ every 15 min for 20 h to 40 h in an Epoch2 reader (BioTek, Agilent) at 37°C with double orbital shaking.

**Autolysis Assay**

The autolysis assay was adapted from [3]. Briefly, strains from overnight cultures in TSB were harvested (1 min at 15871 rcf) and washed once with PBS. Bacteria were adjusted to OD_600_ = 0.1 in 10 ml TSB (in 100 ml flasks) and grown for 5 h at 37°C with agitation. Cells were harvested and washed twice in PBS and once in ice-cold water. The pellet was resuspended in PBS and the OD_600_ was adjusted to 2. From the bacterial suspension 190 µl was transferred to a 96-well plate and 10 µl of 0.5% Triton X100-PBS (corresponds to 0.025% end concentration) was added. Lysis was monitored using OD_578_ for 4 h in an Epoch2 reader (BioTek, Agilent) at 37°C with double orbital shaking.

**Biotin-dependent growth support assay on plates**

Biotin-depleted sTMS agar was prepared as described in the material and method section. To determine the degree of biotin-dependent growth support on plates, bacteria from BHI-T overnight cultures were collected (1 min at 15871 rcf) and washed once with PBS. For *S. aureus ΔbioA* used as a lawn, OD_600_ was adjusted to 0.05 and streaked on plates using sterile cotton swabs. For bacteria grown as spot, the OD_600_ was adjusted to 5 and 5 μl was spotted on plates. 1 μl of 100 μM biotin was used as control and plates were incubated for 48 h at 37°C. Growth diameters were determined by measuring spot and halo diameters using ImageJ.

1. Krismer B, Liebeke M, Janek D, *et al.* Nutrient limitation governs *Staphylococcus aureus* metabolism and niche adaptation in the human nose. *PLoS Pathog* 2014;**10**:e1003862. DOI: 10.1371/journal.ppat.1003862

2. Camus L, Franz J, Gerlach D, *et al.* Tyrosine availability shapes *Staphylococcus aureus* nasal colonization and interactions with commensal communities. 2025. Microbiology, 2025.

3. Biswas R, Martinez RE, Göhring N, *et al.* Proton-binding capacity of *Staphylococcus aureus* wall teichoic acid and its role in controlling autolysin activity. *PLoS ONE* 2012;**7**:e41415. DOI: 10.1371/journal.pone.0041415
